# Immunogenic cell death-related genes as prognostic biomarkers and therapeutic insights in uterine corpus endometrial carcinoma: an integrative bioinformatics analysis

**DOI:** 10.3389/fonc.2025.1588703

**Published:** 2025-07-24

**Authors:** Tianfei Yi, Zhenglun Yang, Peng Shen, Yan Huang

**Affiliations:** ^1^ Department of Monitoring and Early Warning, YinZhou District Center for Disease Control and Prevention, Ningbo, China; ^2^ Department of Gynaecology, YinZhou Third Hospital, Ningbo, China; ^3^ Department of Radiology, The Affiliated People’s Hospital of Ningbo University, Ningbo, China

**Keywords:** immunogenic cell death, uterine corpus endometrial carcinoma, immunotherapy, prognostic model, immune microenvironment

## Abstract

**Introduction:**

Immunogenic cell death (ICD) is the phenomenon in which tumor cells undergo the transition from a non-immunogenic state to an immunogenic state upon their demise as a result of external stimuli. While ICD systems have been widely adopted in oncological research, their specific utilization for Uterine Corpus Endometrial Carcinoma (UCEC) investigations has received comparatively little attention.

**Methods:**

The ICD score was assessed using single-sample gene set enrichment analysis (ssGSEA). Differentially expressed genes (DEGs) were identified from transcriptomic data processed with the "DESeq2" R package. A prognostic model was then developed by integrating these DEGs with clinical variables. The immune landscape was characterized through multiple bioinformatics approaches, and immunotherapy response was predicted using the Tumor Immune Dysfunction and Exclusion (TIDE) algorithm. Additionally, drug sensitivity analysis was performed based on the Genomics of Drug Sensitivity in Cancer (GDSC) database.

**Results:**

In this study, we calculated ICD scores based on 74 ICD-related genes to explore the role of ICD in UCEC progression. We observed that patients with higher ICD scores exhibited a more favorable prognosis, and the score showed a positive correlation with mutation burden (r=0.16, P<0.001). Then we identified 587 upregulated DEGs and 153 downregulated DEGs in high-ICD group compared to low-ICD group. The former was predominantly associated with immune pathways, which was validated in GEO dataset. Using the 64 common DEGs obtained from both TCGA and GEO datasets, we developed a prognostic model specifically tailored for UCEC patients, incorporating five optimal prognostic genes (CD52, SLC30A3, ST8SIA5, STAT1 and TRBC1). Furthermore, the inclusion of clinical factors (stage and ICD score) significantly enhanced the model's predictive ability. The ICD score exhibited positive correlations with immune cell infiltration, as verified by ESTIMATE, xCell, TIMER, MCPcounter, EPIC, and IPS algorithms. Finally, we found that hyper-immunogenicity may be sensitive to immunotherapy and certain drugs (AZD5991, Ibrutinib, Osimertinib, AGI-5198, Savolitinib, Sapitinib, AZ960, AZD3759 and Ruxolitinib), while PCI-34051 and Vorinostat showed sensitivity in patients with hypo-immunogenicity.

**Discussion:**

Our results demonstrate that ICD plays an important role in UCEC progression, suggesting that ICD-related markers could serve as potential targets for prognosis and treatment.

## Introduction

Mammalian cells respond to environmental disturbances by activating signaling pathways that attempt to restore cellular homeostasis. However, when these perturbations exceed cellular repair capacities, the initially cytoprotective signals shift to a cytotoxic mode, ultimately promoting regulated cell death (RCD) (1). A growing number of RCD types have been identified, including necroptosis, pyroptosis, and ferroptosis, among others (2). In 2005, Guido Kroemer et al. discovered that tumor cells dying in response to anthracyclines (such as doxorubicin) can elicit an effective antitumor immune response even without any adjuvant. This response suppresses the growth of inoculated tumors or leads to the regression of established neoplasia (3). This unique form of functionally distinctive RCD is now commonly referred to as immunogenic cell death (ICD).

When tumor cells undergo cell death as a result of external stimulation, the process of transitioning from a non-immunogenic state to an immunogenic state is referred to as ICD (4). Unlike other forms of cell death, ICD triggers the immune system to recognize and selectively target cancerous cells (5). During ICD, dying tumor cells release specific molecules known as damage-associated molecular patterns (DAMPs), including high mobility group protein B1 (HMGB1), ATP, and calreticulin (CRT), among others, into the surrounding environment (6). The release of DAMPs initiates a series of events that promote the recruitment and activation of immune cells, particularly dendritic cells, which play a critical role in initiating an immune response. Dendritic cells capture antigens from dying tumor cells and present them to T cells, stimulating an adaptive immune response specifically targeting the tumor cells (7). Additionally, ICD can induce the release of pro-inflammatory cytokines, which further amplify the immune response and facilitate the recruitment of additional immune cells to the tumor site (1). The immune response activated through ICD is crucial as it enhances the body’s inherent ability to recognize and eliminate tumor cells. This provides a mechanism for the immune system to precisely target and attack cancer cells, potentially leading to more effective anti-tumor responses.

Uterine corpus endometrial carcinoma (UCEC), one of the most prevalent malignancies in the female reproductive system, is an epithelial tumor originating from the endometrial lining (8). According to the 2022 Global Cancer Statistics (GLOBOCAN), UCEC accounted for an estimated 420,242 new cases and 97,704 deaths worldwide, highlighting its significant global burden (9). Traditionally, UCEC has been classified into two distinct subtypes: Type I tumors are estrogen-dependent, predominantly exhibiting endometrioid histology and a more favorable prognosis; whereas Type II tumors are estrogen-independent, frequently demonstrating aggressive serous or clear cell histology and associated with poorer clinical outcomes (10). Recent advances in molecular characterization, particularly through The Cancer Genome Atlas (TCGA) initiative, have redefined the classification of UCEC into four molecular subtypes: *POLE* ultramutated, microsatellite instability-high (MSI-H), copy-number low (endometrioid), and copy-number high (serous-like). Each subtype demonstrates unique clinicopathological characteristics and prognostic implications (11). Notably, *POLE*-mutated tumors, characterized by an ultrahigh mutation burden and enhanced immunogenicity, are associated with exceptional survival outcomes. In contrast, copy-number high serous-like tumors display aggressive biological behavior and intrinsic resistance to standard treatment modalities (12). Although increasing attention has been paid to the mutation (13) and immune microenvironment (14, 15) in UCEC, the role of immunogenic cell death (ICD) in UCEC progression remains largely unclear. In particular, studies exploring ICD-related gene expression patterns and their association with tumor mutation, prognosis, and potential therapeutic sensitivity in UCEC are limited. Thus, elucidating key genes and immunological landscapes is vital to discover prognostic biomarkers and advance precision medicine for UCEC.

In this study, we conducted a comprehensive bioinformatics analysis to investigate the interplay between ICD, tumor mutation burden, and immune microenvironment in UCEC. By leveraging publicly available datasets, we hypothesized that ICD-related genes regulate immune cell infiltration and mutational patterns, thereby influencing prognosis and drug sensitivity. By constructing a prognostic model combining ICD-related genes and clinical factors, we aimed to provide a robust tool for prognostic risk and therapeutic decision-making. Our analysis also explored ICD-associated pathways, immune microenvironment dynamics, and drug sensitivity profiles, bridging the gap between bioinformatics insights and clinical applications.

## Materials and methods

### Data sources and processing

We selected a total of 74 ICD-related genes, after removing duplicates from three different sources: Garg et al. (16) contributed 34 genes, Huang et al. (17) contributed 25 genes, and Xu et al. (18) contributed 32 genes. To conduct our analysis, we obtained transcriptome data from The Cancer Genome Atlas (TCGA) database, which included read count profiles and Fragments Per Kilobase per Million (FPKM) values. Genes with an expression rate lower than 20% were excluded from the analysis. Read count data were employed to perform differential expression analysis using the “DESeq2” R package. The threshold for determining differentially expressed genes (DEGs) was set at a false discovery rate (*FDR*) <0.05. Genes with a fold change (*FC*) > 1 in the high ICD group were considered up-regulated DEGs, while those with an *FC* < 1 were considered down-regulated DEGs. For other analyses, including downstream analysis of mutation data, we utilized the “Maftools” package (19). Additionally, we obtained a validation set (GSE17025) from the NCBI Gene Expression Omnibus (GEO) database to validate our findings.

### Single-sample gene set enrichment analysis

Using the “GSVA”, “GSEABase”, and “limma” R packages, we calculated ICD scores for each sample based on 74 ICD-related genes through the ssGSEA algorithm. Samples were then stratified into high- or low-ICD groups using the median ICD score as the cutoff. Additionally, we applied the same ssGSEA approach to quantify immune checkpoint expression levels and tumor immune microenvironment (TIME) cell infiltration scores.

### Function enrichment analysis and gene set enrichment analysis

To compare the biological themes between the low- and high-ICD cohorts, we performed Gene Ontology (GO) analysis using the Bioconductor package “clusterProfiler” to identify enriched functional categories among DEGs. The R package “ggplot2” was used to visualize the top 10 GO terms and KEGG pathways with the highest enrichment scores with adjusted *P*-value<0.05. Next, we distinguished the signal pathway differences between high and low ICD groups by GSEA software 3.0, and set “c2.cp.kegg.v2022.1.Hs.symbols.gmt” as the reference database. Visualize the top ten differential pathways based on Normalized Enrichment Scores (NES).

### Random intersection generation for GEO-TCGA intersection validation

Through differential analysis, we obtained N up/down-DEGs from the GEO gene set, M up/down-DEGs from the TCGA gene set, and X intersection genes between these two gene sets. To further investigate the significance of the overlap between the GEO and TCGA DEGs, we performed a permutation test. We randomly selected N genes from the GEO gene set and calculated the intersection with the M DEGs from the TCGA gene set. This process was repeated 10,000 times, and we obtained a distribution of the number of intersecting genes (Y). Finally, we applied a *t*-test to compare the observed value of X (the actual number of intersecting genes) with the distribution (Y) obtained from the permutation test. If the *P*-value from the *t*-test was less than 0.05, we considered the difference between X and Y to be statistically significant.

### Characterization of immune landscape

Gene expression data were used to characterize the immune microenvironment of samples, using a variety of bioinformatics tools. ESTIMATE (20) (Estimation of Stromal and Immune cells in Malignant Tumor tissues using Expression data) algorithm was used to evaluate the tumor purity and infiltration of immune/stromal cells: the stromal score, which predicts the presence of stromal cell types in the tumor bulk; the immune score, which infers the infiltration of immune cells in tumor tissue; and the estimate score, which estimates the tumor purity. To obtain a more comprehensive understanding of immune cell composition, 118 tumor microenvironment cells were collected from the “IOBR” package (21). Additionally, validation sets of immune cells were acquired from the xCell (22), TIMER (23), MCPcounter (24), and EPIC (25) platforms. The immunophenoscore (IPS) was used to measure the immune state of the samples. The IPS score utilizes various markers of immune response or immune tolerance to quantify and visualize four different immunophenotypes in a tumor sample: antigen presentation, effector cells, suppressor cells, and checkpoint markers. It also generates a *z-score* that summarizes these four categories. A higher *z-score* indicates a more immunogenic sample (26).

### Prediction of response to immunotherapy

The Tumor Immune Dysfunction and Exclusion (TIDE) algorithm (27) was used to predict the efficacy of immune checkpoint blockade (anti-PD-1/anti-CTLA-4) therapy. The web application, source code and analysis results of TIDE are available at http://tide.dfci.harvard.edu. A *t*-test was used to compare the differences between the responder and non-responder groups.

### Drug sensitivity exploration

The Genomics of Drug Sensitivity in Cancer (GDSC) database, available at www.cancerRxgene.org, is a comprehensive public resource providing information on drug sensitivity in cancer cells and molecular markers associated of drug response (28). To analyze the relationship between drug sensitivity and molecular markers, we obtained IC50 values of drugs and gene expression profiles from the GDSC database using “oncoPredict” algorithm (29). The IC50 value represents the concentration required for 50% inhibition of cell growth in a given cell line. We calculated correlations between ICD scores and both drug IC50 values and drug response-associated target genes. The inverse relationship between ICD score-IC50 value and ICD score-target genes were interpreted as effective triangular feedback loops, indicating that the ICD score influences drug sensitivity, which subsequently affects the IC50 values and target genes associated with drug response.

### Construction of the ICD-related prognostic model

To evaluate the prognosis of UCEC patients, both clinical factors and 64 common DEGs from TCGA and GEO datasets were utilized. The workflow to explore prognostic signatures in UCEC patients involved three steps: (1) Validation using Five-Fold Cross-Validation: A five-fold cross-validation method was employed to assess the robustness of the 64 common DEGs modeling. This technique divides the dataset into five subsets or folds, trains the model on four subsets, and validates it on the remaining subset. This process is repeated five times to ensure reliable results. (2) Construction of the Optimal Gene Model: Initially, univariate *Cox* proportional hazards regression screening was performed on the 64 common DEGs. This analysis helps identify genes with a significant association with patient survival. Subsequently, multivariate *Cox* proportional hazards regression screening was applied to further refine the gene model. Based on the findings from these regression analyses, an optimal gene model was constructed. Using this model, risk scores were calculated for each patient. These risk scores provided an indication of the patient’s likelihood of adverse outcomes. (3) Assessment of Overall Effect: The risk scores obtained from the DEGs-based prognostic model were combined with clinical characteristics. The combined information was then introduced into a multivariate *Cox* proportional hazards regression model to evaluate the overall effect on patient prognosis. The performance of the model was assessed using the concordance index (*C*‐index), which measures the accuracy of survival predictions. A higher *C*-index indicates better predictive ability.

### Statistical analysis

The Hotelling *T^2^* test was performed to analyze the differential expression of ICD-related genes between mutation and normal samples. For counting data, such as categorical variables, the *Chi-square* test was used for analysis. To assess survival outcomes, the Kaplan-Meier *(KM)* method was implemented. The correlations between variables were examined using *Pearson* correlation. When analyzing data from three or more groups, Analysis of Variance analysis (*ANOVA*) was performed, and a *t*-test was employed when comparing data between two groups.

## Results

### Identification of high- and low-ICD groups with ssGSEA

**Figure 1:**
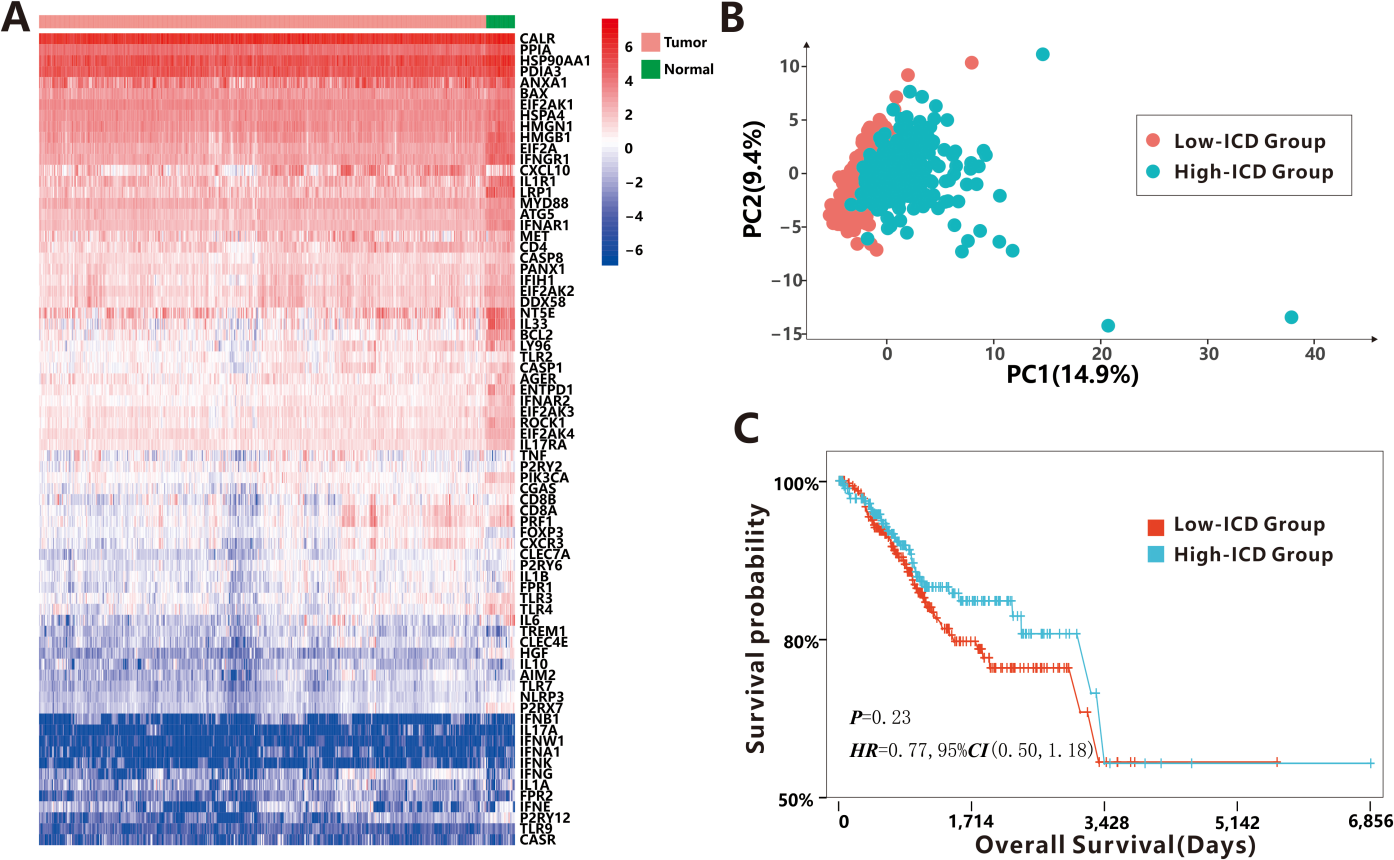
Distinguishability of 74 ICD-related DEGs. **(A)** The expression profiles of 74 ICD-related DEGs between cancer tissues and para-cancerous tissues. **(B)** The PCA between low-ICD group and high-ICD group. **(C)** The survival probability between low-ICD group and high-ICD group.

The study workflow is presented in **Supplementary Figure S1**. Based on the literature and references cited (16–18), a total of 74 ICD-related genes were identified (**Supplementary Table S1**). The study analyzed expression profiles from 543 cancer tissues and 35 para-cancerous tissues obtained from the TCGA-UCEC dataset. Visualization of ICD-related gene expression (**Figure 1A**) revealed significant differences between cancer tissues and para-cancerous tissues as determined by the Hotelling *T^2^* test (*T^2^ =* 15.987, *P*<0.001), indicating that some tumor cells underwent immunogenic transformation, potentially rendering them more susceptible to immune recognition and targeting.

To quantify the level of ICD in each sample, we calculated the ICD score using the ssGSEA algorithm with the 74 ICD-related genes. Samples were then categorized into high-ICD and low-ICD groups based on the median ICD score. Principal Component Analysis (PCA) demonstrated distinct differences in ICD scores between the two groups (**Figure 1B**), indicating that the selected ICD-related genes effectively distinguished samples with high immunogenicity from those with low immunogenicity. However, no significant association was observed between ICD scores and various clinical characteristics such as age, BMI, stage, histological type, new tumor event after initial treatment, and MSI status (**Supplementary Figure S2**). Additionally, although the difference did not reach statistical significance (*P* = 0.23), patients with high ICD scores showed a non-significant trend toward better prognosis compared to those with low ICD scores (**Figure 1C**).

### Association of ICD scores and somatic mutations

**Figure 2:**
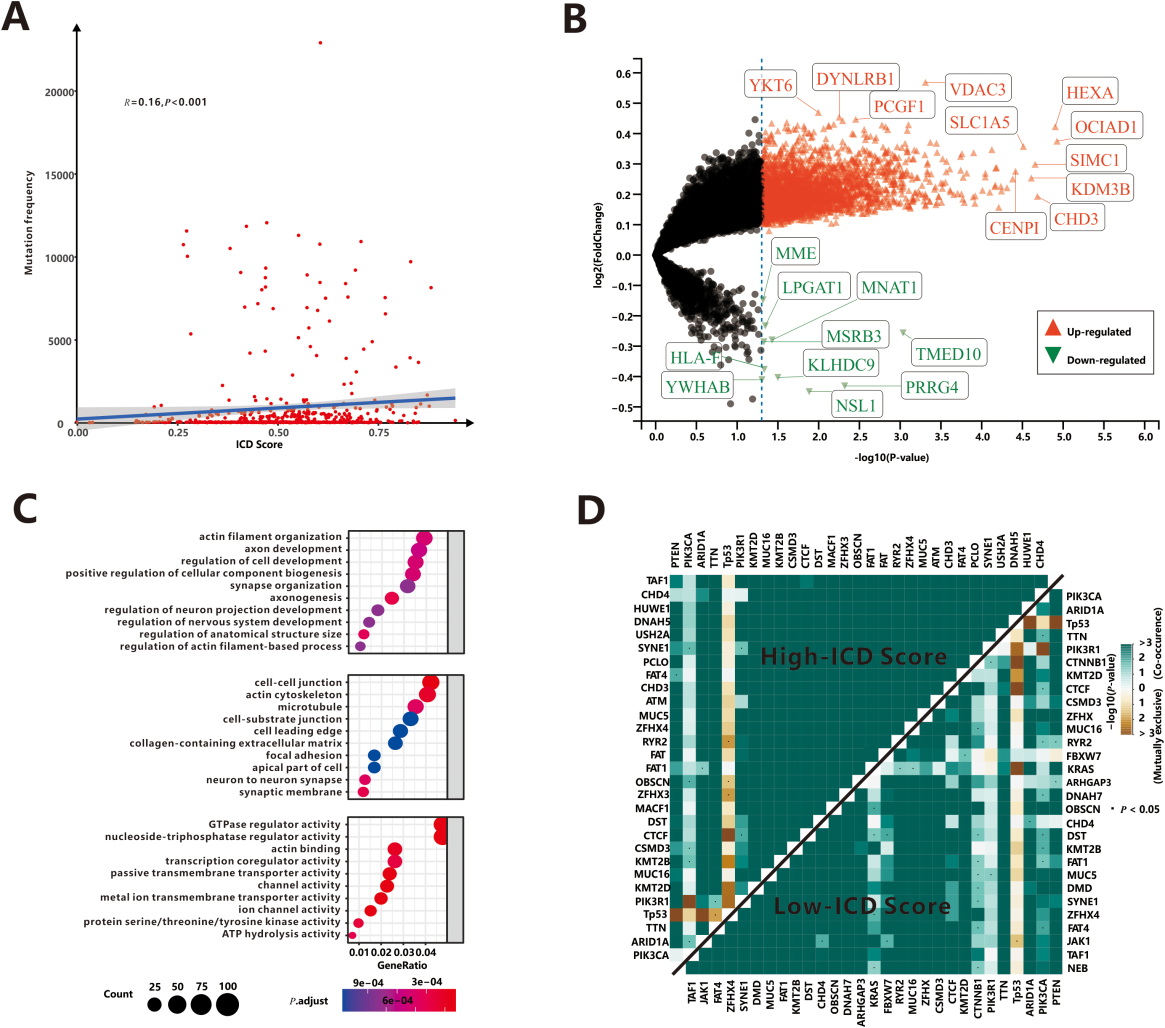
Association of ICD score and somatic mutations. **(A)** The correlation between total mutation frequency and ICD score. **(B)** The mutation genes dysregulated ICD score. **(C)** The GO enrichment functions of mutation genes up-regulated ICD score. **(D)** Mutually exclusive or co-occurring mutations among top 30 mutant genes.

As is well known, somatic mutations (in this study, only non-synonymous mutations were considered.) are frequently encountered in human tumors. Our study investigated the relationship between somatic mutations and ICD scores in tumor cells. The results showed a positive correlation between the total mutation frequency and the ICD score (*r*=0.16, *P*<0.001, **Figure 2A**), indicating that a higher mutation burden is associated with increased immunogenicity. Most classifications of mutations were found to induce immunogenicity in tumor cells (*r*>0.1, *P*<0.05, **Supplementary Figure S3**), except for nonstop mutation (*P*=0.099), in-frame insertions (*P*=0.52) and in-frame deletions (*P*=0.2). To identify specific mutation genes that significantly affected immunogenicity, a *t*-test was performed comparing ICD scores between patients with mutant and wild-type genes (**Figure 2B**). The analysis identified 2,739 mutated genes associated with ICD score upregulation. These genes, such as *VDAC3 etc.*, primarily function in intercellular connections and transmembrane signal transduction mechanisms (**Figure 2C**). Interestingly, only 10 genes, including the MHC Class I complex gene *HLA-F*, were found to be responsible for downregulating ICD scores, potentially suppressing the immune response against tumor cells undergoing immunogenic cell death.

Next, we aimed to identify the top mutation genes that could potentially contribute to these differences. Among the top 30 mutant genes, there were 22 overlapping genes, including *PTEN*, *PIK3CA*, *ARID1A*, *TP53* and *TTN* et al. Previous research by Yeang et al (30). demonstrated that dysregulated pathways often involve mutations in key genes occurring in a mutually exclusive or co-occurring manner. We utilized the somaticInteractions (31), which employed pair-wise Fisher’s exact test to detect significant gene pairs exhibiting co-occurrence or mutual exclusivity, to examine interaction patterns in our cohort. Somatic interactions of high-ICD cohort were significant more than low-ICD cohort (**Figure 2D**). Specifically, there were 380 co-occurrence and 11 mutually exclusive pairs in the high-ICD cohort, while 344 co-occurrence and 9 mutually exclusive pairs were observed in the low-ICD cohort. These findings suggest that patients with hyper-immunogenicity may experience more frequent mutations in a mutually exclusive or co-occurring manner.

### Identification of DEGs and signal pathways between the high- and low-ICD groups

**Figure 3:**
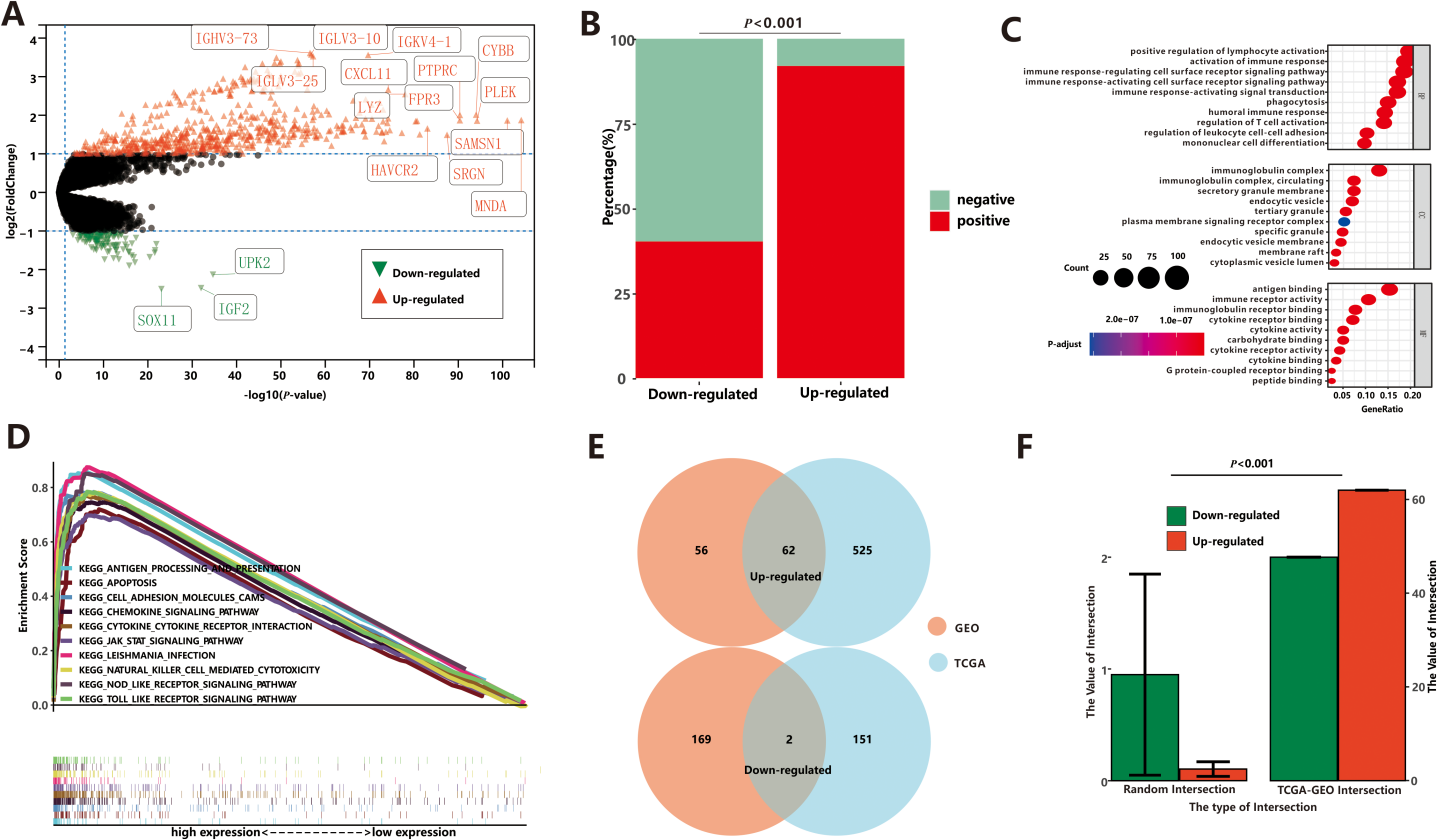
Differential expression analysis between low-ICD group and high-ICD group based on TCGA. **(A)** The DEGs between low-ICD group and high-ICD group. **(B)** Correlation between DEGs and ICD-related genes. **(C)** The GO enrichment functions of up-regulated DEGs. **(D)** The GSEA of up-regulated DEGs. **(E)** The common DEGs between TCGA and GEO. **(F)** Stochastic Verification based on the Permutation Test.

As two ICD groups showed different prognostic tendencies, we aimed to identify key DEGs and signaling pathways in each group in order to explore the biomarkers involved in prognosis modulation. In this study, we identified a total of 587 dysregulated genes that were upregulated and 153 that were downregulated in the high-ICD group compared to the low-ICD group (**Figure 3A**; **Supplementary Table S2**). When examining the relationship between these DEGs and ICD markers, interesting patterns emerged. The majority of the upregulated genes showed significant co-expression with ICD markers, with 17,054 positive and 1,488 negative DEG-ICD relationships, suggesting a strong association between these genes and ICD markers. Conversely, downregulated genes exhibited a mutually exclusive expression pattern with ICD markers, with 422 positive and 626 negative DEG-ICD relationships. *Chi*-square test analysis revealed a significant difference between the up- and down-regulated DEG-ICD relationships (**Figure 3B**, *P*<0.001).

Next, we conducted Gene Ontology (GO) enrichment analysis to gain insights into the functional characteristics of the dysregulated genes in the two ICD groups. Upregulated genes were primarily associated with immune-related processes, including positive regulation of lymphocyte activation, activation of immune response, B cell activation, and other immune system-related processes (**Figure 3C**). On the other hand, the downregulated genes showed enrichment in ion channel activity and neuronal actions, indicating a potential role in neuronal functions. To further investigate the functional implications of the ICD score, we performed Gene Set Enrichment Analysis (GSEA). Results from GSEA were consistent with GO analysis. We observed that pathways associated with a high-ICD score were predominantly related to immune pathways (**Figure 3D**). These included cytokine-cytokine receptor interaction, nod-like receptor signaling pathway, natural killer cell-mediated cytotoxicity, among others. These findings reinforce the notion that a high ICD score is closely associated with immune-related processes and pathways.

To validate our findings, we analyzed microarray expression profiles from 79 endometrial cancer patients (GEO dataset), stratified into high- and low-ICD groups based on ICD scores. The high-ICD group showed 118 upregulated and 171 downregulated DEGs (**Supplementary Figure S4A**, **Supplementary Table S2**). Association analyses revealed that most upregulated genes positively correlated with ICD markers (1,342 positive vs. 69 negative relationships), while downregulated genes showed stronger negative correlations (1,058 positive vs. 1,512 negative relationships), with statistically significant differences (**Supplementary Figure S4B**, *P*<0.001). Gene Ontology enrichment analysis (**Supplementary Figure S4C**) and GSEA (**Supplementary Figure S4D**) further demonstrated that the upregulated genes were primarily associated with the immune system. These results from both the TCGA and GEO databases confirm the strong robustness of ICD-related DEGs.

Finally, the intersections between the TCGA and GEO datasets were depicted in **Figure 3E**. Among these intersections, there were 62 commonly upregulated genes and 2 commonly downregulated gene. To assess the reliability, we conducted 10,000 randomized interactions between GEO and TCGA datasets and compared them with our actual datasets. As illustrated in **Figure 3F**, the actual overlap was significantly higher than randomized expectations (*P*<0.05), confirming the analysis’s validity and enhancing confidence in the common DEGs.

### Construction and validation of the prognostic model based on ICD-related DEGs

It is evident that DEGs associated with immunogenicity can significantly impact cancer-related processes in UCEC patients. Leveraging the 64 common DEGs derived from the TCGA and GEO datasets, we developed a prognostic model specifically tailored to UCEC patients. This model holds great potential in predicting outcomes and guiding treatment decisions.

To assess the predictive performance of the 64 common DEGs, we employed a 5-fold cross-validation method. The dataset was divided into five randomly selected subsets, with four subsets used for training and the remaining subset for validation. This process was repeated five times, with each subset serving as the validation set once. The results (**Supplementary Table S3**) demonstrated that the training models showed mediocre performance, with as average concordance index (*C-*index) of 0.668. The average *AUC* values for the training sets were 0.639 (1-year), 0.685 (3-year), and 0.733 (5-year), while the corresponding values for the testing sets were 0.660 (1-year), 0.697 (3-year), and 0.745 (5-year). These findings demonstrate the robustness of the prognostic model, as the performance of the testing sets closely mirrored that of the training sets.

**Figure 4:**
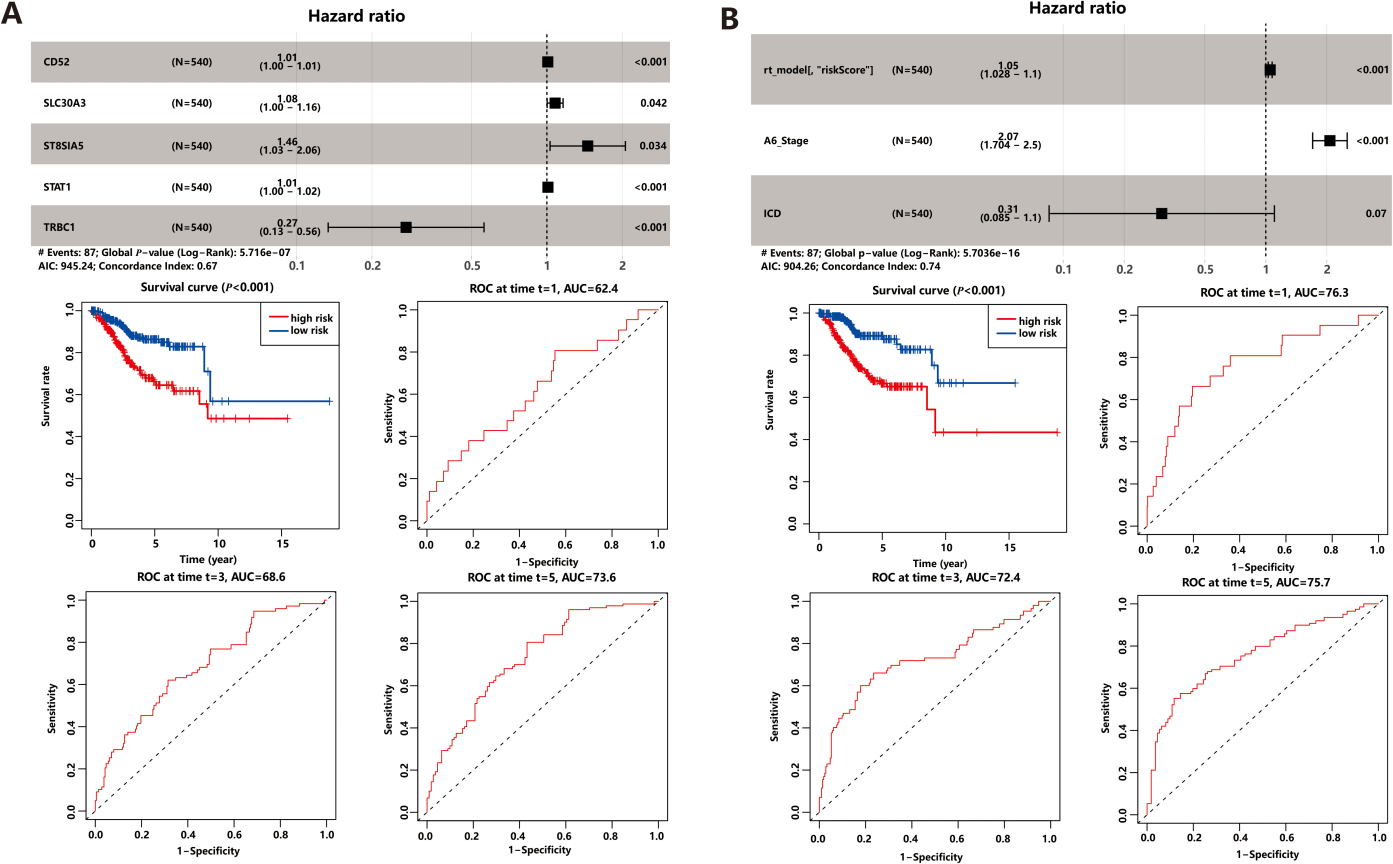
Prognostic Model. **(A)** Gene model based on ICD-related DEGs. **(B)** Comprehensive model based on gene model and clinical factors.

To identify possible prognostic biomarkers, we performed univariate *Cox* regression analysis of 64 common DEGs, and genes with *P*<0.05 were selected for multivariate *Cox* proportional hazards regression to obtain optimal prognostic models. The gene model we derived consisted of five optimal prognostic immune-related genes (*CD52*, *SLC30A3*, *ST8SIA5*, *STAT1*, and *TRBC1*), where all genes except *TRBC1* was identified as a risk factor (**Figure 4A**). Then the risk score of each patient was calculated by the gene model and all patients were divided into high-risk and low-risk groups based on the median risk score. The results, depicted in scatterplots and heat maps (**Supplementary Figure S5A**), showed that high-risk patients had a tendency for earlier mortality compared to low-risk patients. Furthermore, *KM* survival analysis revealed that overall survival was significantly poorer in the high-risk group compared to the low-risk group (*P*< 0.001, **Figure 4A**). The prognostic model demonstrated predictive performance, with area under the curve (*AUC*) values of 0.624 (1-year), 0.686 (3-year), and 0.736 (5-year), suggesting its potential as a prognostic tool for UCEC patients.

Prognostic DEGs demonstrated powerful predictive performance only at fifth year in both cross-validation and gene model. In order to enhance the predictive power of the prognostic model, we integrated clinical factors, such as age, BMI, MSI score, stage, and ICD score, into the gene model. Univariate and multivariate *Cox* regression analysis identified three prognostic markers in the comprehensive model: the gene model risk score, disease stage, and ICD score (**Figure 4B**). Among these markers, the risk score and stage were found to be risk factors, while the ICD score acted as a protective factor. *KM* survival analysis (**Figure 4B**) and scatterplots (**Supplementary Figure S5B**) showed that the comprehensive risk score had high predictive power. Interestingly, the comprehensive model demonstrated even better prediction performance compared to the gene model alone. The area under the curve (*AUC*) values for the comprehensive model were 0.763 for 1-year survival, 0.724 for 3-year survival, and 0.757 for 5-year survival, indicating improved discriminatory power across different time points. Additionally, the inclusion of clinical factors significantly enhanced the model’s discrimination ability. The *C-index*, which measures the model’s discriminatory power, increased to 0.743 for the comprehensive model, whereas it was 0.665 for the gene model alone. This implies that the comprehensive model, which combines gene expression data with relevant clinical factors, provides more accurate prognostic predictions.

### Evaluation of the association of ICD score and tumor microenvironment

**Figure 5:**
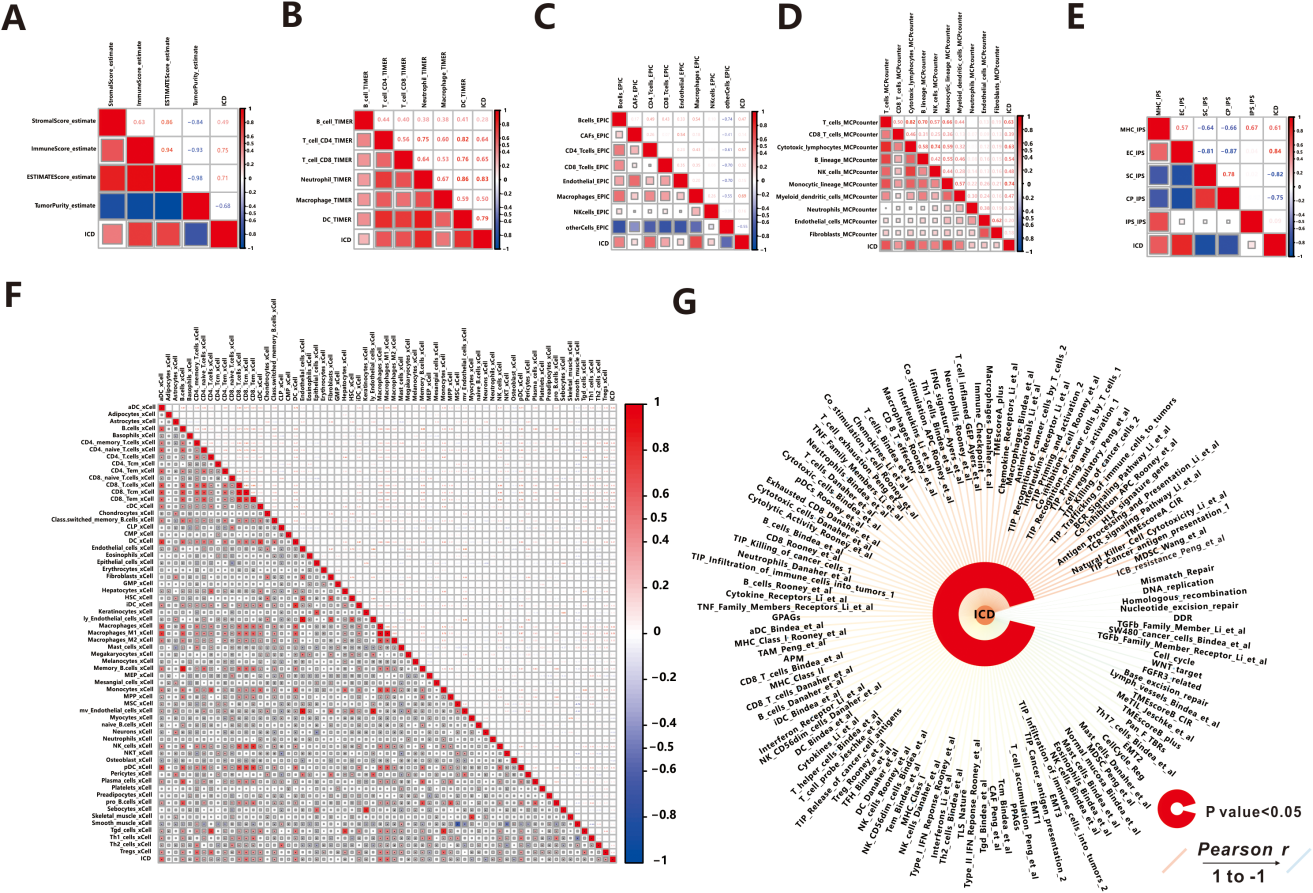
Association between ICD score and Tumor Microenvironment. **(A)** ESTIMATE. **(B)** TIMER. **(C)** EPIC. **(D)** MCPcounter. **(E)** IPS. **(F)** xCell. **(G)** IOBR.

It is widely recognized that the immune response plays a crucial role in the process of ICD (1), such as in Head and Neck Squamous Cell Carcinoma (32). Owing to the tight correlation between the process of ICD process and immune-related biological pathways, we conducted further investigations to explore the link between the ICD score and tumor-infiltrating immune cells. Initially, we utilized the ESTIMATE algorithm to quantify the overall infiltration of immune cells. The results revealed a positive correlation between the ICD score and Stromal Score, Immune Score, and ESTIMATE Score, while a negative correlation was observed between the ICD score and Tumor Purity (**Figure 5A**). This indicates that patients with active ICD are more likely to develop anti-tumor immunity, and lower tumor purity is associated with better prognosis (33). To validate these findings, we performed ssGSEA analysis to assess 118 types of tumor microenvironment cells selected from the “IOBR” package (21). Out of these, 110 cells showed significant correlations with the ICD scores, with 108 demonstrating positive associations and 2 displaying negative associations (**Figure 5G**). It is worth noting that the majority of the positively related cells were involved in the immune microenvironment, while the remaining cells were not directly associated with immunity, indicating that patients with more frequent ICD had a more active immune response.

Then we explored the relationship between 64 kinds of immune cells and the ICD score utilizing the xCell algorithm (**Figure 5F**). In detail, the ICD score was positively correlated with the majority of immune cells, such as B cells, CD4 T cells, CD8 T cells, Macrophages, Monocytes, NK cells, Neutrophils, DC cells, Endothelial cells, and others, which were the primary effector cells and Antigen presentation cells in the anti-tumor immune response. The consistent results were obtained from TIMER (**Figure 5B**), MCPcounter (**Figure 5C**), and EPIC (**Figure 5D**) algorithms, indicating extensive infiltration of immune cells (such as B cells, CD4 T cells, CD8 T cells, Macrophages, Neutrophils, DC cells, NK cells, Monocytic, and Endothelial cells) in patients with active ICD. Additionally, we found that ICD stimulated the aggregation of fibroblasts in MCPcounter analysis. Fibroblasts play a crucial role in the tumor microenvironment, influencing tumor progression through modulation of immune response and promotion of tumor growth and invasion (34). Furthermore, we utilized the immunophenoscore (IPS) method to evaluate the immune state of the samples (**Figure 5E**). Notably, patients with active ICD exhibited increased expression of MHC molecules and effector cells involved in the anti-tumor immune response, while showing lower expression of suppressor cells and immune checkpoint molecules that may suppress the immune response. Moreover, patients with higher levels of active ICD demonstrated higher IPS *z-*scores, indicating a more immunogenic population (35).

### Effect of ICD on immunotherapy response and drug sensitivity

**Figure 6:**
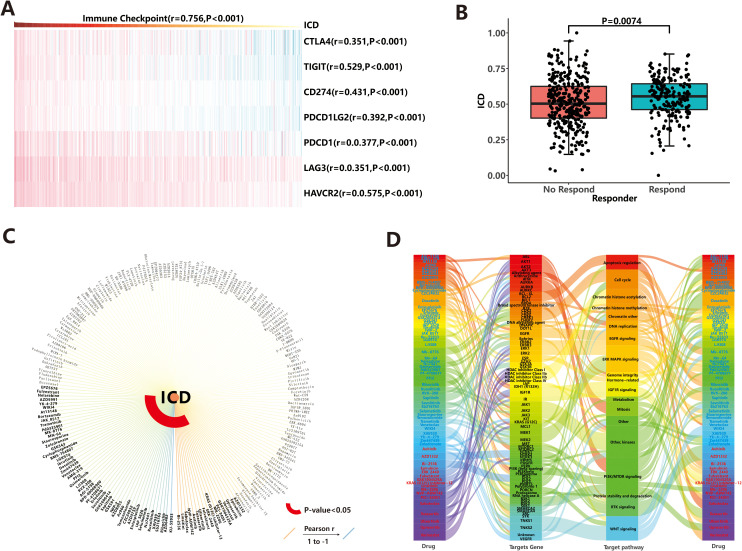
Drug response prediction. **(A)** Correlation between immune checkpoints and ICD score. **(B)** Immunotherapeutic efficacy based on TIDE web tool. **(C)** Chemotherapeutic agents and molecular-targeted drugs based on GDSC database. **(D)** Targeting pathways for sensitive drugs.

In recent years, immunotherapy has attracted significant attention due to its ability to enhance the body’s natural immune response against tumors. It works by blocking immune checkpoint (ICP) ligand-receptor binding, thereby reactivating T cell activity and promoting endogenous anti-tumor immunity (36). In our study, we specifically selected seven common immune checkpoints to predict the response to immunotherapy, and the expression values of all seven genes showed a positive correlation with the ICD score (*r*>0.3, *P*<0.001, **Figure 6A**). Next, we calculated the immune checkpoint score using the ssGSEA algorithm and explored the correlation between the ICP score and the ICD score. As expected, a higher ICP score was associated with stronger immunogenicity (*r*=0.756, *P*<0.001), suggesting that immunotherapy may be a preferred treatment option for patients with a high ICD score. To assess the potential response to immunotherapy for each sample, we utilized the TIDE web tool. The analysis revealed that out of the total sample size, 204 patients (37.6%) exhibited a positive response to immunotherapy, while 339 patients (62.4%) did not respond favorably (**Figure 6B**). Additionally, the ICD scores were significantly higher (*P*=0.007) in the response group (mean: 0.545 ± 0.141) compared to the no-response group (mean: 0.508 ± 0.172). This suggests that patients with stronger immunogenicity, as indicated by higher ICD scores, are more likely to benefit from immunotherapy.

In addition to immunotherapy, chemotherapeutic agents and molecular-targeted drugs are important in cancer therapy. In our study, we screened 198 compounds from the GDSC database to identify immunogenicity-related drugs. The results showed that IC50 values of 69 drugs were significantly associated with ICD score (**Figure 6C**; **Supplementary Table S4**). Among these drugs, patients with high ICD score showed sensitivity to 52 drugs, including KU-55933, AZD6482, and others, while those with low ICD score were sensitive to 17 drugs (such as BI-2536, Linsitinib, et al.). Notably, nine of these drugs belonged to the class of PI3K/MTOR inhibitors, which exert anti-tumor effects by targeting the PI3K/MTOR signaling pathway (**Figure 6D**). This highlights the importance of PI3K/MTOR-related targets in tumor therapy. Additionally, other common targetable pathways for drug involvement in anti-cancer activities include ERK MAPK signaling, WNT signaling, and kinases (**Figure 6D**). To evaluate the efficacy of these 69 drugs more accurately, we explored the triangular feedback loops among ICD score, drugs, and target genes. The results revealed that 11 drugs formed triangular feedback loops. For example, PCI-34051 and Vorinostat inhibited HDAC family genes and demonstrated anti-tumor effects, particularly in patients with hypo-immunogenicity (**Figure 6D**), where HDAC family genes were highly expressed (**Supplementary Figure S6**). On the other hand, drugs like AZD5991, Ibrutinib, Osimertinib, AGI-5198, Savolitinib, Sapitinib, AZ960, AZD3759 and Ruxolitinib were sensitive to patients with hyper-immunogenicity (**Figure 6D**), and their target genes (including *MCL1*, *BTK*, *EGFR*, *IDH1*, *MET*, *JAK1*, *JAK2*, *JAK3*, *etc.*) were generally highly expressed in the hyper-immunogenic state (**Supplementary Figure S6**). These findings suggest that the mentioned drugs can be used to guide the treatment of patients with endometrial carcinoma, taking into account their immunogenicity profiles.

## Discussion

ICD constitutes a prominent pathway for the activation of the immune system against cancer, which determines the prognosis of patients and the efficacy of anti-cancer therapies to some extent. The application of ICD features has already shown promise in predicting the prognosis and guiding treatment decisions for various tumors such as gastric cancer (37), head and neck squamous cell carcinoma (32), and high-grade glioma (38). In this UCEC-focused study, we conducted a comprehensive analysis to investigate the important role of ICD. First, we calculated the ICD score for each sample using the ssGSEA algorithm based on 74 ICD markers and stratified samples into high- or low-ICD groups according to the median of the ICD score. This allowed us to explore the relationship between the ICD score and various molecular events, including DNA variation, gene expression, and the tumor microenvironment. Through this exploration, we aimed to uncover potential mechanisms underlying the occurrence and development of UCEC. Additionally, we utilized ICD-related DEGs to construct a prognostic model. By incorporating clinical data, we aimed to enhance the robustness of the model and improve their predictive accuracy for patient outcomes. Lastly, we used the ICD scores obtained from our analysis to guide the treatment of patients diagnosed with UCEC. This approach holds promise for tailoring treatment strategies to individual patients based on their ICD profiles, potentially leading to improved therapeutic outcomes.

In this study, we observed that UCEC patients with high ICD scores exhibited both a higher frequency of mutations and increased immune cell infiltration. Mutations can alter amino acid coding sequences, leading to tumor expression of mutant proteins that are absent in normal cells. These abnormal protein sequences serve as neoantigens, which can be recognized by T cells and trigger an immune response against tumor cells. The presentation of neoantigens occurs through the major histocompatibility complex (MHC, also known as human leukocyte antigen (HLA) in humans) molecules on the tumor cell surface, and these cells are immunogenic (39). Furthermore, we found that different mutations vary in their immunogenicity. Insertion/deletion and frameshift mutations were particularly immunogenic due to their significant alterations in the amino acid sequence and spatial structure. These types of mutations have a stronger binding affinity to MHC molecules, increasing the likelihood of being recognized as neoantigens by T cells.

Indeed, the immune response plays a crucial role in the process of ICD. In our study, we also discovered a positive correlation between ICD scores and immune cell infiltration in UCEC patients. Yoshihara et al. described an ESTIMATE method to assess the score of stromal and immune cells in tumor samples, and the estimate score was positively related to tumor purity (20). In our study, patients with hyper-immunogenicity tended to have higher ESTIMATE scores and lower tumor purity, and lower tumor purity means better prognosis (33), which could explain why these UCEC patients were associated with favorable prognosis. During ICD, dying cells release specific molecules known as damage-associated molecular patterns (DAMPs). These DAMPs act as “danger signals” and are recognized by antigen-presenting cells (APCs), such as dendritic cells (DCs) (40). Subsequently, activated DCs migrate to lymphoid organs, like lymph nodes, where they present the processed antigens to T cells (41). The interaction between major histocompatibility complex (MHC)-antigen complexes on dendritic cells and T cell receptors (TCRs) on T cells leads to the activation of specific T cell subsets, including CD8+ cytotoxic T cells and CD4+ helper T cells (42). Activated T cells then undergo proliferation and differentiation into effector cells, which can directly kill target cancer cells and generate anti-tumor immunity (43).

Furthermore, the expression of immune checkpoint molecules on immune cells inhibits immune cell function, thereby preventing the body from mounting effective anti-tumor immune responses. These “checkpoints” may be exploited by tumors to facilitate immune evasion within tumor tissue (44). Immunotherapy works by reactivating anti-tumor immunity through the blockade of immune checkpoint pathways. In our study, we discovered a positive correlation between immune checkpoint expression score and ICD score in UCEC patients. This suggests that patients with higher levels of immunogenicity also exhibit increased expression of immune checkpoint molecules. Importantly, based on predictions made from the TIDE web platform (45), we anticipated that patients with high ICD score would demonstrate a favorable response to immunotherapy. This indicates that individuals with stronger immunogenicity could derive greater benefits from immunotherapeutic treatments. Our findings align with previous research that has demonstrated the potential of immunotherapy in treating hyper-immunogenic cancers, such as colon cancer (46) and head-and-neck squamous cell cancer (32).

Not all patients with hyper-immunogenicity are suitable for immunotherapy due to individual differences and the risk of severe side effects (47). Therefore, it is necessary to explore alternative treatment options. In our study, we used the “oncoPredict” algorithm to estimate the susceptibility of 198 compounds screened from the GDSC database. We found that 69 drugs showed a significant association with ICD scores. Among these drugs, AZD5991, Ibrutinib, Osimertinib, AGI-5198, Savolitinib, Sapitinib, AZ960, AZD3759, and Ruxolitinib demonstrated sensitivity in patients with hyper-immunogenicity. These drugs target genes such as *MCL1*, *BTK*, *EGFR*, *IDH1*, *MET*, *JAK1*, *JAK*2, and *JAK3*, which are generally highly expressed in hyper-immunogenicity. Ruxolitinib, for instance, is an oral selective *JAK1/JAK2* inhibitor that has gained FDA approval for treating certain conditions. Recent studies have shown that Ruxolitinib can enhance the immunogenicity of cancer cells and promote ICD in various cancer types, including diffuse large B-cell lymphoma (DLBCL) (48). It achieves this by increasing the production of cytokines and chemokines that attract immune cells to the tumor microenvironment, thereby facilitating antigen presentation and T-cell activation (49, 50). Ruxolitinib also upregulates MHC class I expression on cancer cells, further facilitating T-cell recognition and activation (51). AZ960, which is also a *JAK2* inhibitor, has shown promising results in inducing growth arrest and apoptosis in adult T-cell leukemia cells (52). Furthermore, *EGFR* tyrosine kinase inhibitors (TKIs) such as AZD3759, Sapitinib, and Osimertinib have been found to enhance ICD and increase the expression of DAMPs, including calreticulin and HMGB1 (53). These DAMPs play a crucial role in promoting dendritic cell maturation and T cell activation. AZD-5991, a highly selective inhibitor of *MCL1*, may participate in the mechanism that induces ICD by affecting the metabolic pathways of SphK and S1P (54). Although specific studies on the relationship between Savolitinib, AGI-5198, Ibrutinib and ICD are lacking, our results suggest that these inhibitors may be effective for patients with hyper-immunogenicity in UCEC. It is important to note that while most immunogenic drugs are effective for high immunogenicity, there are exceptions. For example, PCI-34051 and Vorinostat, which are effective for low immunogenicity, inhibit HDAC family genes. HDAC family genes play a role in inhibiting cancer cell apoptosis and promoting cancer angiogenesis (55). Inhibitors targeting HDAC offer a potential therapeutic strategy for cancer by affecting proliferation, differentiation, angiogenesis, and migration.

The identification of prognostic biomarkers plays a crucial role in predicting patient outcomes and guiding personalized treatment decisions. In this study, we conducted a comprehensive analysis to identify prognostic biomarkers and develop a predictive model for a specific condition. The results demonstrated the significant impact of combining gene expression data with clinical factors in improving the accuracy of prognostic predictions. The resulting gene model consisted of five immune-related genes, including four risk factors (*CD52*, *SLC30A3*, *ST8SIA5*, and *STAT1*) and one protective factor (*TRBC1*). These five genes each exhibit mechanistic links to ICD or UCEC progression through immune-related pathways. CD52, a glycoprotein highly expressed on lymphocytes and dendritic cells, may facilitate immune cell interactions within the tumor microenvironment (TME) or modulate immune synapse formation during antigen presentation (56), potentially influencing the efficiency of ICD-induced immune responses in UCEC. SLC30A3 (ZnT3) regulates zinc transport, and zinc homeostasis is intricately linked to various cell death modalities and immune function (57). Aberrant expression of SLC30A3 may disrupt intracellular zinc homeostasis, exacerbate oxidative stress, and contribute to epithelial damage and tumor immune evasion in endometrial tissue. ST8SIA5 encodes a sialyltransferase (58) that modifies glycan structures on the tumor surface, and this glycosylation remodeling may shield UCEC cells from immune surveillance, limiting ICD-mediated immune activation. STAT1, a well-established immune-related transcription factor, is frequently activated in endometrial carcinoma (59) and plays a critical role in upregulating MHC class I molecules and mediating interferon-induced antitumor responses (60)—both essential components of ICD signaling. In contrast, TRBC1, identified as a protective factor, is integral to T-cell receptor diversity and antigen recognition. Higher expression of TRBC1 may indicate preserved cytotoxic T-cell function and enhance tumor cell elimination through ICD-associated adaptive immunity (61). Moreover, incorporating both the gene risk score and relevant clinical factors (risk factors: Stage; protective factor: ICD score) in the comprehensive model provides more accurate prognostic predictions.

While our study provides novel insights into immunogenic cell death (ICD)-related biomarkers and therapeutic implications in uterine corpus endometrial carcinoma (UCEC), several limitations must be acknowledged. First, the entire analysis was conducted in silico based on publicly available retrospective datasets, including TCGA and GEO. Although these databases are widely used and well-curated, their reliance may introduce inherent biases (e.g., sample selection, batch effects) and limit the generalizability of our findings to broader or more diverse patient populations (62). Second, although we performed internal validation of our prognostic model using five-fold cross-validation and confirmed DEG patterns across TCGA and GEO datasets, we acknowledge the lack of an independent external validation cohort. Without external clinical validation, there remains a potential risk of model overfitting, which could overestimate its real-world predictive performance (63). Future studies incorporating prospective UCEC cohorts or independent multicenter datasets are needed to further confirm the robustness and clinical utility of our model. Third, our study did not incorporate any *in vitro* or *in vivo* experimental validation of the key findings. Specifically, the expression and biological function of the identified prognostic ICD-related genes, the immune landscape differences, and the predicted drug sensitivities have not yet been validated through laboratory assays such as RT-qPCR, immunohistochemistry, or drug response experiments in UCEC models. While such experiments are beyond the scope of this bioinformatics study, we fully recognize their importance and have highlighted them as critical next steps for future translational research. Finally, the predictions of drug sensitivity were based solely on correlative analysis using the GDSC database and the oncoPredict algorithm. These predictions, while valuable for hypothesis generation, do not account for the full complexity of tumor-drug interactions *in vivo* and should be interpreted with caution. Mechanistic studies and pharmacological validation in experimental UCEC models are warranted to verify the efficacy and relevance of these candidate compounds. In summary, while our study offers a comprehensive and integrative computational framework for exploring ICD in UCEC, further experimental and clinical investigations are essential to validate, refine, and translate these findings into practical clinical applications.

## Conclusion

Our research emphasizes the significant role of ICD in UCEC, highlighting its correlation with DNA mutations, gene expression, immune cell infiltration, and its potential for improving prognosis and targeted therapy. These results are expected to provide valuable support and guidance for more effective clinical practice and treatment recommendations.

## Data Availability

The original contributions presented in the study are included in the article/**Supplementary Material**. Further inquiries can be directed to the corresponding author/s.

## Glossary

ICD: Immunogenic Cell Death
UCEC: Uterine Corpus Endometrial Carcinoma
ssGSEA: Single-sample Gene Set Enrichment Analysis
DEGs: Differentially Expressed Genes
GDSC: Genomics of Drug Sensitivity in Cancer
RCD: Regulated Cell Death
DAMPs: Damage-associated Molecular Patterns
HMGB1: High Mobility Group Protein B1
CRT: Calreticulin
TCGA: The Cancer Genome Atlas
FPKM: Fragments per Kilobase per Million
FDR: False Discovery Rate
FC: Fold Change
GEO: Gene Expression Omnibus
GO: Gene Ontology
GSEA: Gene Set Enrichment Analysis
NES: Normalized Enrichment Scores
ESTIMATE: Estimation of Stromal and Immune cells in Malignant Tumor tissues using Expression data
IPS: Immunophenoscore
TIDE: Tumor Immune Dysfunction and Exclusion
C‐index: Concordance index
KM: Kaplan-Meier
ANOVA: Analysis of Variance Analysis
PCA: Principal Component Analysis
AUC: Area Under the Curve
ICP: Immune Checkpoint
HLA: Human Leukocyte Antigen
APCs: Antigen-presenting Cells
DCs: Dendritic Cells
MHC: Major Histocompatibility Complex.
TCRs: T cell receptors
DLBCL: Diffuse Large B-cell Lymphoma
TKIs: Tyrosine Kinase Inhibitors
MPNs: Myeloproliferative Neoplasms
TIME: Tumor immune microenvironment.
