# Supplementary material for: Immunogenic cell death-related genes as prognostic biomarkers and therapeutic insights in uterine corpus endometrial carcinoma: an integrative bioinformatics analysis
Source: Front Oncol. 2025 Jul 24;15:1588703. doi: 10.3389/fonc.2025.1588703 (PMC12328154; doi:10.3389/fonc.2025.1588703)
Supplement: Supplementary file 1 [file DataSheet1.docx]

**Supplementary Figures**

**
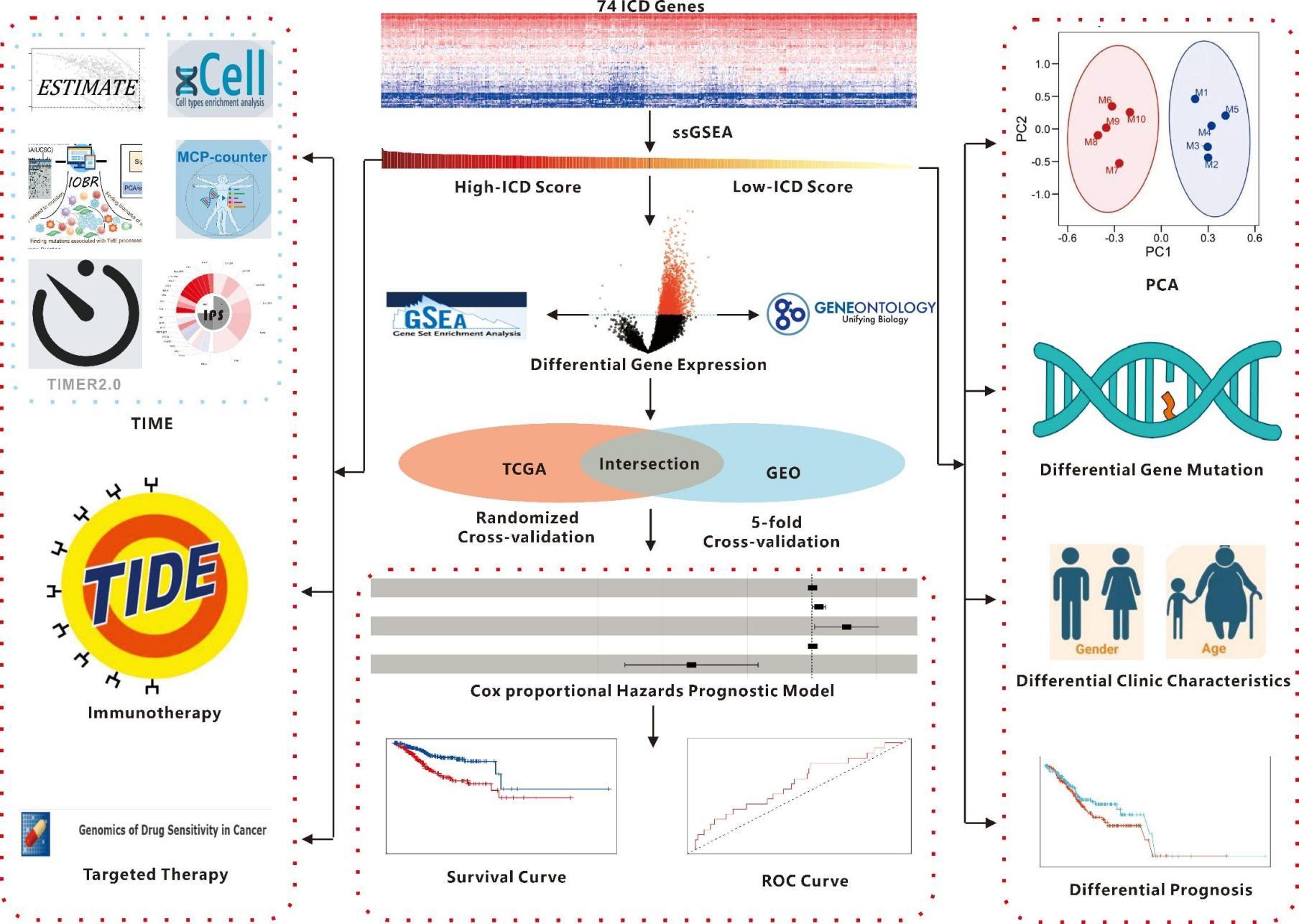
**

**Figure S1 Flow Chart of this Study.**


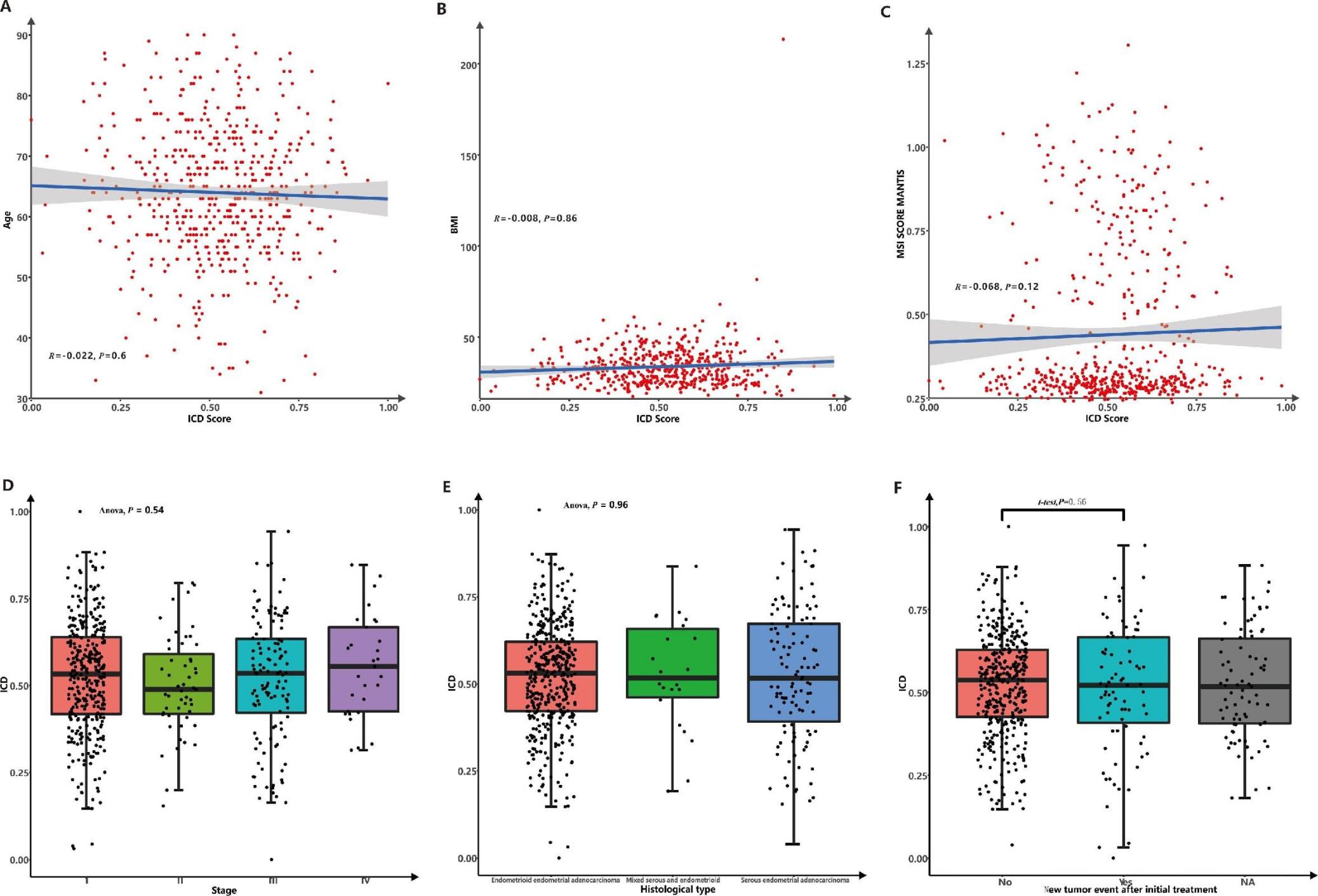


**Figure S2 Association between ICD score and clinical characteristics.** A Pearson correlation between age and ICD score. B Pearson correlation between BMI and ICD score. C Pearson correlation between MSI score and ICD score. D ANOVA among stage I-IV. E ANOVA among three histological types. F T-test between free and new tumor event after initial treatment.


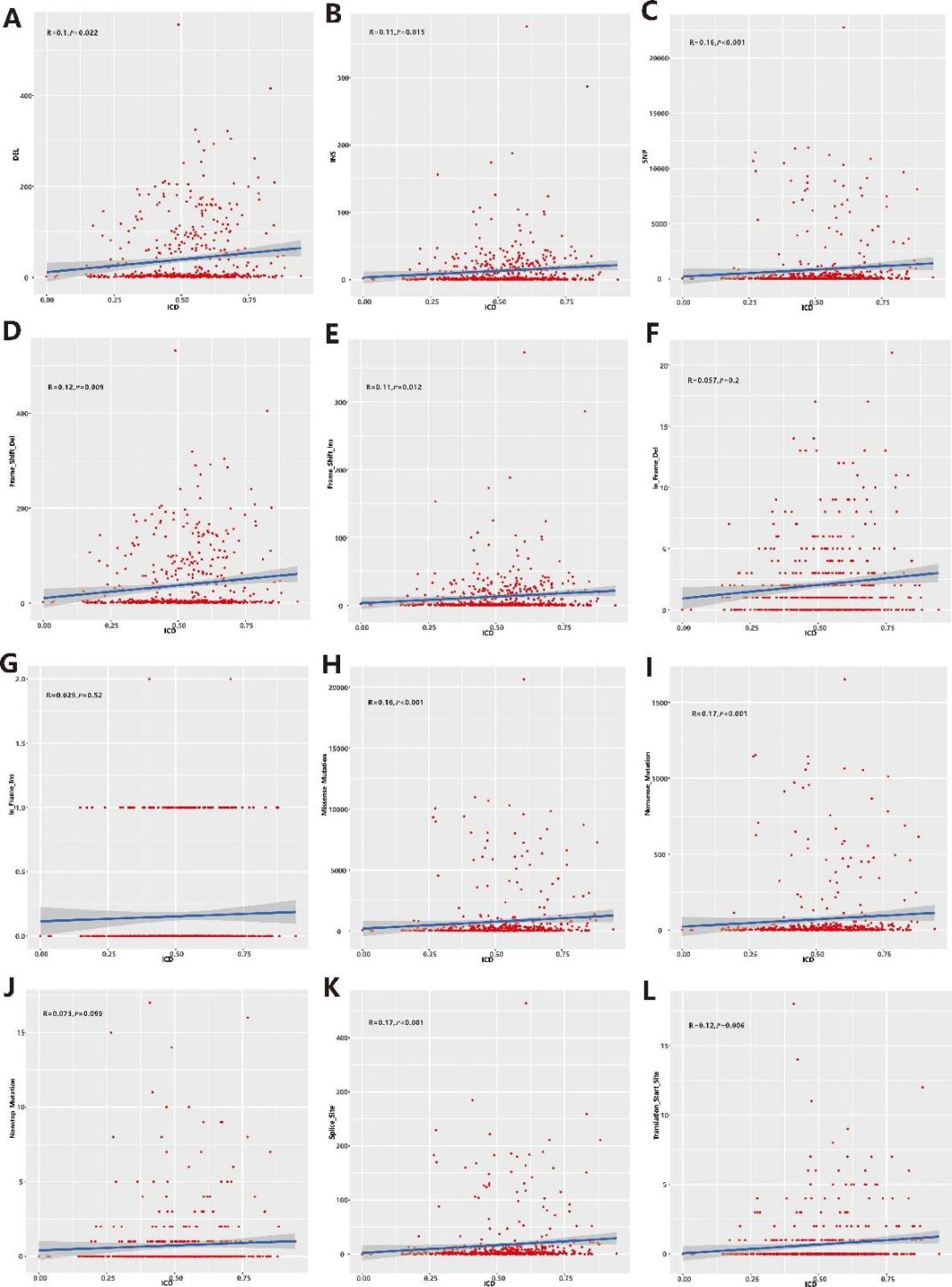


**Figure S3 Association between ICD score and different type of mutation.** A DEL. B INS. C SNP. D Frame_Shift_Del. E Frame_Shift_Ins. F In_Frame_Del. G In_Frame_Ins. H Missense_Mutation. I Nonsense_Mutation. J Nonstop_Mutation. K Splice_Site. L Translation_Start_Site.


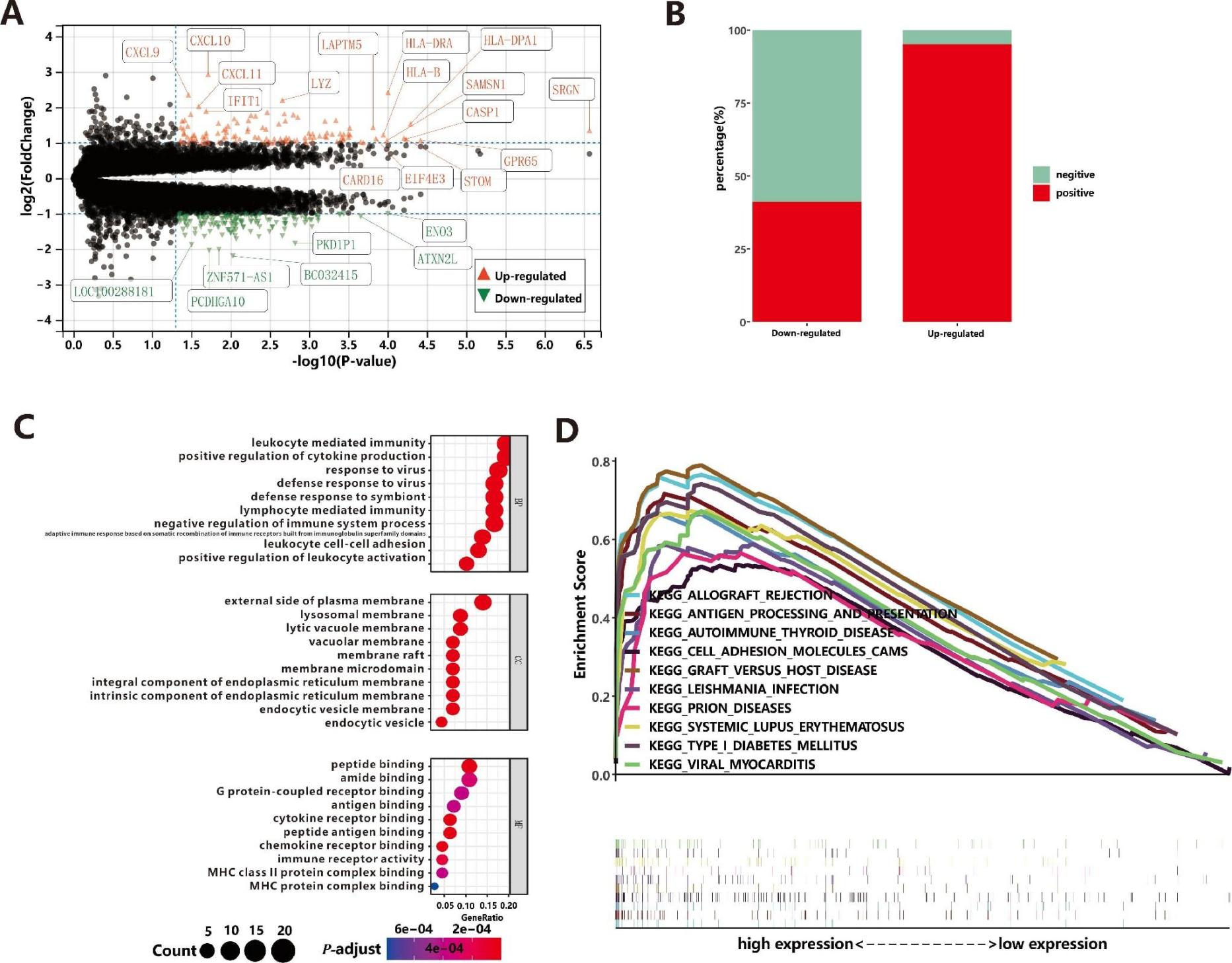


**Figure S4 Differential expression analysis between low-ICD group and high-ICD group based on GSE17025.** A The DEGs between low-ICD group and high-ICD group. B Correlation between DEGs and ICD-related genes. C The GO enrichment functions of up-regulated DEGs. D The GSEA of up-regulated DEGs.


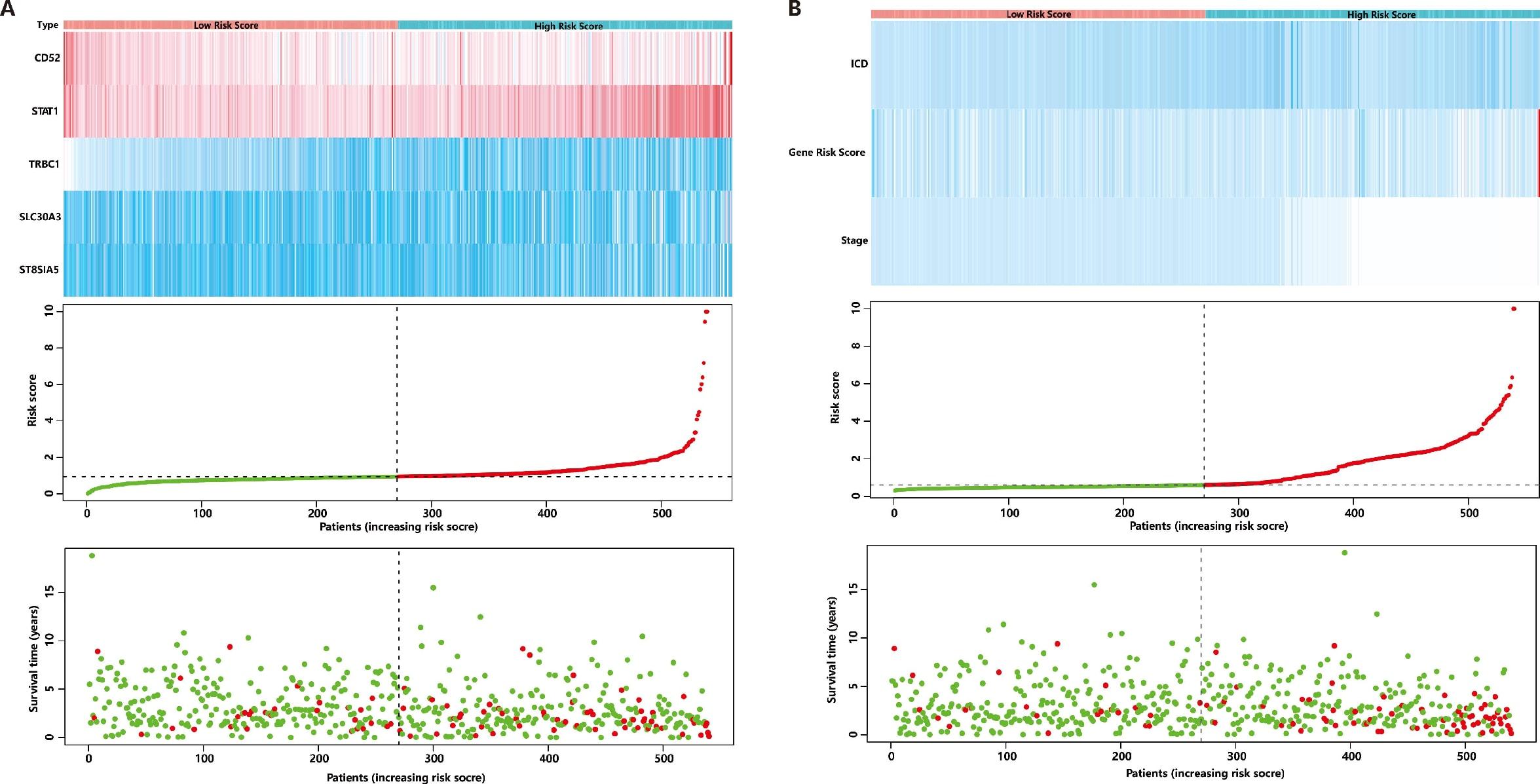


**Figure S5 Validation of the robustness of prognostic models.** Heatmap in the top panel showing the gradient of the optimal prognostic IRGs. The color in the heatmap represents the normalized values of log2FPKM. The scatterplots in the middle and bottom panels respectively illustrate the distribution of survival status and risk score of UCEC patients. A Gene model based on ICD-related DEGs. B Comprehensive model based on gene model and clinical factors.


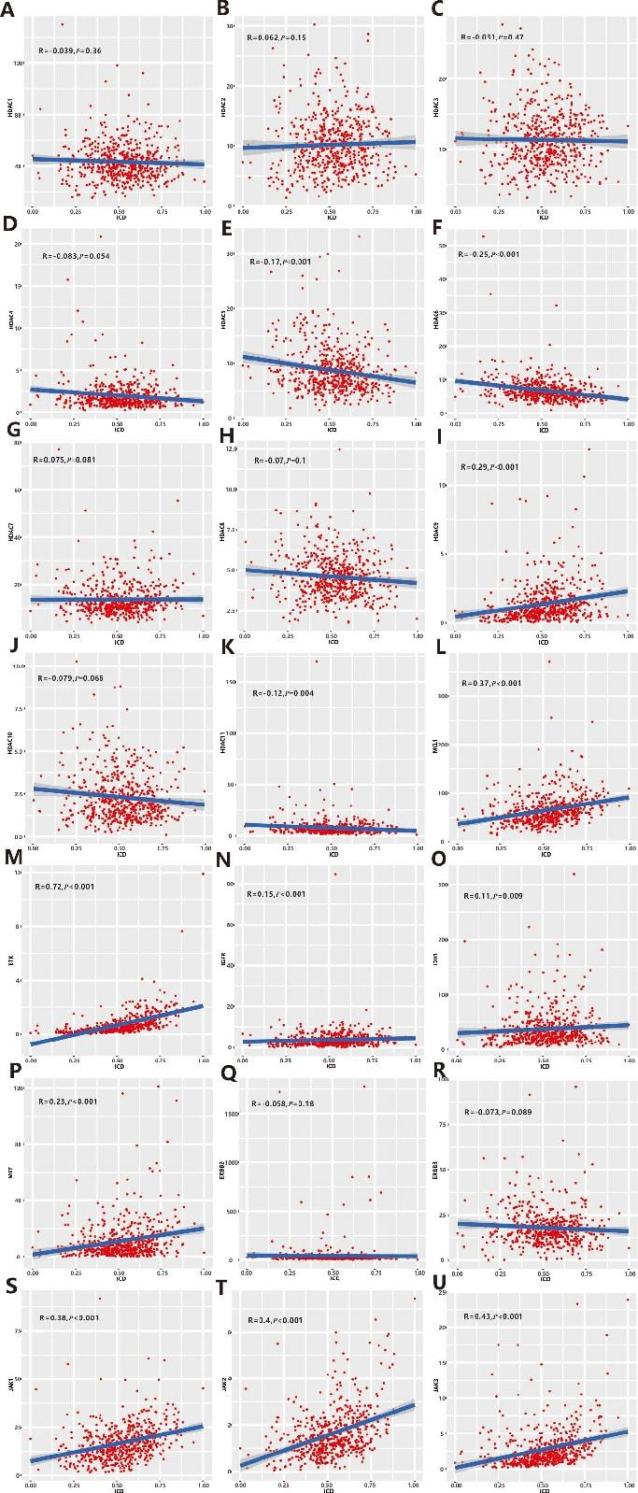


**Figure S6 The correlation between ICD scores and targeted genes of 11 drugs forming triangular feedback loops.**

**Supplementary Tables**

**Table S1 Immunogenic cell death-related genes**

| Abhishek et al. | Huang et al. | Xu et al. | Abhishek et al.\|Huang et al. | Abhishek et al.\|Xu et al. | Huang et al.\|Xu et al. | Abhishek et al.\|Huang et al.\|Xu et al. |
| --- | --- | --- | --- | --- | --- | --- |
| ATG5 | LRP1 | AGER | EIF2AK3 | HSP90AA1 | FPR1 | CALR |
| BAX | EIF2A | AIM2 | IFNA1 | NLRP3 | PANX1 | HMGB1 |
| CASP1 | ANXA1 | BCL2 | IFNB1 |  | P2RY2 | P2RX7 |
| CASP8 | IFNE | CASR |  |  | TLR3 | TLR4 |
| CD4 | IFNK | CGAS |  |  |  |  |
| CD8A | IFNW1 | CLEC4E |  |  |  |  |
| CD8B | CXCL10 | CLEC7A |  |  |  |  |
| CXCR3 | IFNAR1 | DDX58 |  |  |  |  |
| ENTPD1 | IFNAR2 | FPR2 |  |  |  |  |
| FOXP3 | HGF | HMGN1 |  |  |  |  |
| IFNG | MET | HSPA4 |  |  |  |  |
| IFNGR1 | EIF2AK2 | IFIH1 |  |  |  |  |
| IL10 | EIF2AK1 | IL1A |  |  |  |  |
| IL17A | EIF2AK4 | IL33 |  |  |  |  |
| IL17RA |  | P2RY12 |  |  |  |  |
| IL1B |  | P2RY6 |  |  |  |  |
| IL1R1 |  | PPIA |  |  |  |  |
| IL6 |  | ROCK1 |  |  |  |  |
| LY96 |  | TLR2 |  |  |  |  |
| MYD88 |  | TLR7 |  |  |  |  |
| NT5E |  | TLR9 |  |  |  |  |
| PDIA3 |  | TREM1 |  |  |  |  |
| PIK3CA |  |  |  |  |  |  |
| PRF1 |  |  |  |  |  |  |
| TNF |  |  |  |  |  |  |

**Table S2 Differentially expressed genes**

| TCGA Up-regulated genes | TCGA Down-regulated genes | GEO Up-regulated genes | GEO Down-regulated genes |
| --- | --- | --- | --- |
| FGR | CACNA2D2 | ADAMDEC1 | AC005306.3 |
| CD38 | HHATL | AIF1 | ADM5 |
| ITGAL | F7 | APOL1 | AF279780 |
| NOS2 | CAMK2B | ASPN | AFG3L1P |
| TRAF3IP3 | SEZ6 | BST2 | AK090844 |
| ETV7 | SNAP91 | C16orf54 | AKAP10 |
| CD4 | PDZD4 | C1QA | ALDH8A1 |
| BTK | SLC12A3 | C1QB | ALOX12P2 |
| TYROBP | TRPM5 | C1S | ALX4 |
| LTF | CYP2W1 | C3 | ANKHD1 |
| ALOX5 | PAX2 | CALM1 | ANKRD20A11P |
| CD6 | ACTN2 | CARD16 | ANKRD36 |
| WAS | MYH7B | CASP1 | ANKRD36B |
| MARCO | CHRNA3 | CCL18 | ANKZF1 |
| CYP24A1 | MT3 | CCL5 | AP000347.2 |
| BIRC3 | DLL3 | CD14 | AQPEP |
| TYMP | COL9A3 | CD163 | ATAD5 |
| SLAMF7 | CRTAC1 | CD2 | ATHL1 |
| TNFRSF1B | SEZ6L | CD3G | ATXN2L |
| STAP1 | NTSR1 | CD52 | BC022892 |
| MSR1 | ST8SIA5 | CD74 | BC032415 |
| LCP2 | ZFR2 | CHL1 | BC041025 |
| TNFRSF17 | UPK1A | CLEC5A | BHMT |
| TNFRSF9 | ATP4A | CPA3 | CACNA1D |
| TNIP3 | TMEM59L | CRISPLD1 | CAPN12 |
| CHI3L2 | GCK | CSF2RB | CCDC14 |
| CD84 | CDH23 | CTSO | CCDC150 |
| SPI1 | FGF8 | CX3CR1 | CCDC152 |
| DAPP1 | EBF3 | CXCL10 | CDK10 |
| FCGR2B | NKX3-2 | CXCL11 | CEACAM19 |
| TBX21 | DDX25 | CXCL9 | CEP164 |
| ARHGAP15 | CCKBR | DDX60 | CHTF18 |
| ICAM3 | UPK2 | DIRAS2 | CLTC-IT1 |
| APBB1IP | CUX2 | DPYD | COL27A1 |
| CST7 | SLC30A3 | EIF4E3 | CRIPAK |
| P2RY10 | CHD5 | ENPP2 | CT62 |
| SP140 | DLGAP3 | EPB41L3 | DCUN1D2 |
| PTPRC | ACTL8 | EVI2A | DQ570835 |
| FYB1 | APOA1 | EVI2B | EIF3C |
| FCN1 | SFRP5 | FAM26F | EMC3-AS1 |
| CEACAM6 | DBH | FGL2 | EME2 |
| SIGLEC1 | CHST8 | FHL2 | ENO3 |
| SIRPG | HIF3A | FPR3 | ENTPD2 |
| P2RX7 | PIWIL1 | GBP1 | ERV3-1 |
| OAS1 | TCF15 | GIMAP2 | FAM149A |
| LAG3 | F10 | GIMAP4 | FLJ22184 |
| RGS1 | KCNC1 | GNB4 | FLJ35934 |
| ICAM1 | PLPPR3 | GNG2 | FLJ45482 |
| LYZ | GALNT8 | GPR65 | GLYATL1 |
| CD209 | GFAP | GSPT2 | GNG13 |
| IL12RB1 | BARX1 | GZMA | GOLGA8A |
| OSM | DUSP26 | GZMK | HMMR-AS1 |
| CYTH4 | GABBR2 | HCP5 | HSF4 |
| LGALS2 | TUBB2B | HLA-B | IL17RB |
| APOL1 | PIK3C2G | HLA-DPA1 | ITIH4 |
| GRAP2 | FBN3 | HLA-DPB1 | JPX |
| NCF4 | IGLON5 | HLA-DQB1 | KIAA0485 |
| CSF2RB | PRDM16 | HLA-DRA | KIAA0895L |
| IL2RB | ILDR2 | HLA-DRB6 | KIFC2 |
| GZMH | CASQ1 | HLA-F | KNTC1 |
| GZMB | FLG | HLA-G | KRTAP19-3 |
| TCL1A | TAGLN3 | HLA-J | LINC00202-1 |
| MMP9 | GFRA3 | HTR2B | LINC00342 |
| SLA2 | CACNA1B | ICOS | LINC00597 |
| SLCO4A1 | KIRREL3 | IDO1 | LINC00691 |
| SIRPB1 | TLCD3B | IFI27 | LINC00930 |
| HCK | HMGA2 | IFI44L | LINC01000 |
| TLR8 | FMN2 | IFI6 | LINC01125 |
| CD40LG | CELF3 | IFIH1 | LOC100130429 |
| TNFSF13B | FNDC5 | IFIT1 | LOC100272216 |
| CORO1A | TMEM190 | IFIT3 | LOC100288181 |
| IL21R | AZGP1 | ISOC1 | LOC100289019 |
| AQP9 | PTGER1 | ITGB2 | LOC100289092 |
| CD37 | CELF5 | LAP3 | LOC100505874 |
| LILRB1 | NTNG1 | LAPTM5 | LOC100506236 |
| LILRA1 | KCNF1 | LIPA | LOC100506459 |
| RASAL3 | NKX6-1 | LYZ | LOC100507006 |
| EBI3 | CAMKV | MAOB | LOC100652999 |
| CEACAM4 | ZMAT4 | MNDA | LOC101060510 |
| SIGLEC8 | SLITRK5 | MRGPRF | LOC101927166 |
| CD79A | LRFN5 | MS4A4A | LOC101927305 |
| NKG7 | RET | MS4A7 | LOC101927330 |
| CD33 | CRABP1 | MX1 | LOC101928283 |
| CEACAM5 | IGF2 | NAP1L2 | LOC102725116 |
| LILRB5 | TTYH1 | NKG7 | LOC145837 |
| SLC5A5 | KLK4 | OAS2 | LOC284837 |
| PIK3CG | KCNJ4 | OLFML3 | LOC399491 |
| TFEC | GNG4 | P2RY13 | LOC400965 |
| P2RX1 | UGT3A2 | PLAC8 | LOC441124 |
| CCL8 | SERPINA6 | PLSCR1 | LOC494150 |
| ODAM | TRH | PSMB8 | LOC644794 |
| CRTAM | DAB1 | PSMB9 | LOC646014 |
| MS4A6A | KCNH6 | PTGER2 | LOC647070 |
| MS4A4A | IGDCC3 | PTGER4 | LOC692247 |
| IL10RA | SPSB4 | PTPRC | LOC729732 |
| SLC15A3 | CALCB | RGS1 | LUC7L |
| CD5 | TSPEAR | RTN1 | MAMDC4 |
| POU2AF1 | SOX11 | RTP4 | MGC12488 |
| CD69 | LY6H | SAMD9 | MIR3916 |
| CLEC2B | TMEM151B | SAMSN1 | MLPH |
| SELPLG | SHISA3 | SERPING1 | MROH2A |
| BIN2 | DAND5 | SGMS2 | MST1 |
| OAS2 | USH1G | SLAMF8 | MSTN |
| IFNG | KCNIP1 | SLC15A3 | NBEAL1 |
| CLEC4A | C2CD4C | SLC9A3R1 | NDUFB2-AS1 |
| KLRB1 | CNTN2 | SPARCL1 | NKAPP1 |
| VNN2 | MUC6 | SRGN | NKTR |
| LY86 | NTF3 | STAT1 | NPIPA1 |
| GZMK | NAT8L | STOM | OBSCN |
| ITK | C1QL4 | SYNC | PABPC1L |
| CD86 | ERAS | TCEAL7 | PAQR6 |
| IL1A | PTPRT | TGFBR2 | PCDHGA10 |
| CYTIP | ELAVL3 | TMEM200A | PCP2 |
| STAT1 | SLC35F1 | TRBC1 | PDC |
| GNLY | ELOVL2 | TREM2 | PDCD6 |
| IL1RL1 | ZNF536 | UBASH3A | PGF |
| IL18RAP | PCDHA1 | UBE2L6 | PKD1P1 |
| KYNU | SYCE1L | VCAM1 | PLEKHA8P1 |
| PLEK | ENSG00000206549 |  | PMFBP1 |
| NCF2 | TRIM71 |  | PMS2L2 |
| CD2 | MEG3 |  | PMS2P8 |
| SLAMF1 | GCGR |  | POLE |
| CD48 | FXYD7 |  | PPFIA4 |
| PLA2G2D | LINC02593 |  | PPP1R1A |
| GBP1 | PRRT4 |  | PROCA1 |
| FASLG | ENSG00000230699 |  | RAB11FIP3 |
| HSD11B1 | TSPEAR-AS1 |  | RAB40A |
| MUC5B | RPS27P25 |  | RAB6A |
| SPP1 | ARHGDIG |  | RAP1GAP2 |
| IFIT3 | LINC00461 |  | RASEF |
| IFIT2 | FLJ12825 |  | RECQL4 |
| CD274 | LINC02381 |  | RHPN1 |
| TASL | MAGEL2 |  | RP1-199J3.7 |
| CSTA | ENSG00000258010 |  | RP1-202O8.3 |
| CD80 | TMEM179 |  | RP11-108K3.2 |
| CCRL2 | CTXND1 |  | RP11-157P1.4 |
| CCR2 | ENSG00000260293 |  | RP11-216L13.19 |
| TMEM156 | SCX |  | RP11-250B2.3 |
| SASH3 | ENSG00000260816 |  | RP11-301O19.1 |
| LAX1 | CCER2 |  | RP11-334C17.5 |
| CD244 | LINC01977 |  | RP11-357G3.2 |
| LY9 | MIR4737 |  | RP11-421E14.2 |
| SRGN | ENSG00000265638 |  | RP11-674P19.2 |
| ARHGAP9 | ENSG00000266701 |  | RP11-690I21.2 |
| NCKAP1L | ENSG00000270168 |  | RP11-95O2.1 |
| TNFAIP6 | ENSG00000271384 |  | RP13-20L14.1 |
| ZNF831 | ENSG00000272384 |  | RP13-436F16.1 |
| ZBP1 | GRIN2B |  | RP4-647C14.3 |
| CXCL6 | ENSG00000274373 |  | RPPH1 |
| TMEM255A | FLJ16779 |  | RRS1-AS1 |
| IL1B | ENSG00000276863 |  | SCT |
| S1PR4 | ENSG00000278909 |  | SEPSECS-AS1 |
| FFAR2 | ENSG00000280156 |  | SEPT7P2 |
| HCST |  |  | SGK494 |
| CCR7 |  |  | SLC25A34 |
| EVI2A |  |  | SLC26A10 |
| ADGRE2 |  |  | SLC30A3 |
| FGL2 |  |  | SMA4 |
| APOL3 |  |  | SNRK-AS1 |
| RAC2 |  |  | SPDYE2 |
| CPA4 |  |  | SSBP3-AS1 |
| CD68 |  |  | ST8SIA5 |
| SIGLEC9 |  |  | STAG3L3 |
| ARHGEF6 |  |  | SYMPK |
| LBP |  |  | TGM1 |
| APOC1 |  |  | TIGD3 |
| KLHDC7B |  |  | YJEFN3 |
| LSP1 |  |  | ZCWPW1 |
| GMFG |  |  | ZNF141 |
| LILRB2 |  |  | ZNF571-AS1 |
| IDO1 |  |  | ZNF692 |
| FCRLA |  |  |  |
| JCHAIN |  |  |  |
| CLEC10A |  |  |  |
| FCRL2 |  |  |  |
| ALOX5AP |  |  |  |
| CHIT1 |  |  |  |
| EPSTI1 |  |  |  |
| PLAAT4 |  |  |  |
| GIMAP6 |  |  |  |
| GIMAP4 |  |  |  |
| ADAMDEC1 |  |  |  |
| CD180 |  |  |  |
| PTPN22 |  |  |  |
| RSAD2 |  |  |  |
| SAA2 |  |  |  |
| IL2RA |  |  |  |
| DOCK2 |  |  |  |
| KLRD1 |  |  |  |
| KLRC1 |  |  |  |
| TCN1 |  |  |  |
| HAVCR2 |  |  |  |
| OASL |  |  |  |
| TESPA1 |  |  |  |
| STX11 |  |  |  |
| NPL |  |  |  |
| DOCK10 |  |  |  |
| LCP1 |  |  |  |
| IL6 |  |  |  |
| AOAH |  |  |  |
| MYO1G |  |  |  |
| BLK |  |  |  |
| IL1RN |  |  |  |
| SIT1 |  |  |  |
| IRF4 |  |  |  |
| SLCO2B1 |  |  |  |
| MMP7 |  |  |  |
| CASP1 |  |  |  |
| IFI44L |  |  |  |
| DUSP5 |  |  |  |
| CXCL9 |  |  |  |
| CD27 |  |  |  |
| GPR84 |  |  |  |
| GPR65 |  |  |  |
| WARS1 |  |  |  |
| PSTPIP1 |  |  |  |
| BCL2A1 |  |  |  |
| ITGAX |  |  |  |
| IRF8 |  |  |  |
| PIK3R5 |  |  |  |
| MYO1F |  |  |  |
| SIGLEC10 |  |  |  |
| PADI1 |  |  |  |
| C1orf162 |  |  |  |
| CD53 |  |  |  |
| XCL2 |  |  |  |
| FCGR2A |  |  |  |
| FCRL5 |  |  |  |
| S100A8 |  |  |  |
| PTPN7 |  |  |  |
| GZMA |  |  |  |
| TIMD4 |  |  |  |
| FGD2 |  |  |  |
| SCML4 |  |  |  |
| GPR174 |  |  |  |
| IL2RG |  |  |  |
| DOK2 |  |  |  |
| LCN2 |  |  |  |
| FERMT3 |  |  |  |
| MMP3 |  |  |  |
| FCGR1A |  |  |  |
| CD226 |  |  |  |
| RGS18 |  |  |  |
| IL18 |  |  |  |
| AKR1C2 |  |  |  |
| TDO2 |  |  |  |
| KCNK13 |  |  |  |
| CD96 |  |  |  |
| ADGRF1 |  |  |  |
| ADGRF4 |  |  |  |
| CD8A |  |  |  |
| GBP5 |  |  |  |
| LY96 |  |  |  |
| SAMSN1 |  |  |  |
| SLC7A7 |  |  |  |
| VSIG4 |  |  |  |
| SLA |  |  |  |
| BATF |  |  |  |
| CXCL13 |  |  |  |
| MS4A1 |  |  |  |
| XDH |  |  |  |
| CD1E |  |  |  |
| NCF1 |  |  |  |
| SLAMF8 |  |  |  |
| FCER1G |  |  |  |
| C1QC |  |  |  |
| UBASH3A |  |  |  |
| ITGB2 |  |  |  |
| JAML |  |  |  |
| CD3G |  |  |  |
| CCR5 |  |  |  |
| FCRL3 |  |  |  |
| HK3 |  |  |  |
| SIGLEC11 |  |  |  |
| SCIMP |  |  |  |
| LAPTM5 |  |  |  |
| GBP2 |  |  |  |
| GBP4 |  |  |  |
| GFI1 |  |  |  |
| VCAM1 |  |  |  |
| NLRP3 |  |  |  |
| SLAMF6 |  |  |  |
| FCGR3B |  |  |  |
| NEURL3 |  |  |  |
| CTSS |  |  |  |
| TNFAIP8L2 |  |  |  |
| S100A9 |  |  |  |
| S100A12 |  |  |  |
| CXCR1 |  |  |  |
| EOMES |  |  |  |
| TRAT1 |  |  |  |
| MNDA |  |  |  |
| PYHIN1 |  |  |  |
| AIM2 |  |  |  |
| CTLA4 |  |  |  |
| ICOS |  |  |  |
| CD200R1 |  |  |  |
| CXCL5 |  |  |  |
| CXCL1 |  |  |  |
| CPA3 |  |  |  |
| CCR1 |  |  |  |
| LIPH |  |  |  |
| TAGAP |  |  |  |
| TMEM71 |  |  |  |
| CYBB |  |  |  |
| NCF1C |  |  |  |
| PRKCB |  |  |  |
| CLEC4E |  |  |  |
| B2M |  |  |  |
| C15orf48 |  |  |  |
| MS4A7 |  |  |  |
| MEI1 |  |  |  |
| GNGT2 |  |  |  |
| SNX20 |  |  |  |
| CD3D |  |  |  |
| LAIR1 |  |  |  |
| LY6D |  |  |  |
| TMIGD2 |  |  |  |
| CD300C |  |  |  |
| CD300A |  |  |  |
| BATF2 |  |  |  |
| CX3CR1 |  |  |  |
| XIRP1 |  |  |  |
| TAP1 |  |  |  |
| RHOH |  |  |  |
| IL7R |  |  |  |
| SIGLEC7 |  |  |  |
| CXCL10 |  |  |  |
| CXCL11 |  |  |  |
| RNASE2 |  |  |  |
| PTAFR |  |  |  |
| RNASE6 |  |  |  |
| CXCL8 |  |  |  |
| CD52 |  |  |  |
| HTRA4 |  |  |  |
| GPR183 |  |  |  |
| ITGAM |  |  |  |
| GPR25 |  |  |  |
| CD14 |  |  |  |
| MZB1 |  |  |  |
| OSCAR |  |  |  |
| CEACAM3 |  |  |  |
| FPR2 |  |  |  |
| FPR1 |  |  |  |
| GPR34 |  |  |  |
| C3AR1 |  |  |  |
| CXCR6 |  |  |  |
| CLEC7A |  |  |  |
| CLEC12A |  |  |  |
| IL16 |  |  |  |
| CTSW |  |  |  |
| THEMIS |  |  |  |
| CCL19 |  |  |  |
| PARP15 |  |  |  |
| ABCD2 |  |  |  |
| C1QB |  |  |  |
| C1QA |  |  |  |
| OLR1 |  |  |  |
| SAA1 |  |  |  |
| XCR1 |  |  |  |
| CD7 |  |  |  |
| TLR10 |  |  |  |
| CMKLR1 |  |  |  |
| ADGRE1 |  |  |  |
| GPR171 |  |  |  |
| LRRC25 |  |  |  |
| GAPT |  |  |  |
| ZNF683 |  |  |  |
| KCNA3 |  |  |  |
| SAMD9L |  |  |  |
| ZBED2 |  |  |  |
| CD163 |  |  |  |
| CD28 |  |  |  |
| GIMAP7 |  |  |  |
| HLA-DQB1 |  |  |  |
| CIITA |  |  |  |
| TMEM150B |  |  |  |
| FUT7 |  |  |  |
| PRF1 |  |  |  |
| BHLHE22 |  |  |  |
| CXCR2 |  |  |  |
| HLA-V |  |  |  |
| CCL13 |  |  |  |
| P2RY13 |  |  |  |
| TIGIT |  |  |  |
| P2RY8 |  |  |  |
| C1S |  |  |  |
| NCF1B |  |  |  |
| CSF1R |  |  |  |
| LCK |  |  |  |
| MCEMP1 |  |  |  |
| CCR4 |  |  |  |
| SH2D1A |  |  |  |
| CLECL1 |  |  |  |
| IKZF1 |  |  |  |
| EVI2B |  |  |  |
| C16orf54 |  |  |  |
| CD300LF |  |  |  |
| BTLA |  |  |  |
| CD300E |  |  |  |
| ARHGAP30 |  |  |  |
| CXCR3 |  |  |  |
| LILRB4 |  |  |  |
| LILRA5 |  |  |  |
| AKR1C1 |  |  |  |
| FPR3 |  |  |  |
| DMBT1 |  |  |  |
| PDCD1 |  |  |  |
| SELL |  |  |  |
| DPYD |  |  |  |
| CALHM6 |  |  |  |
| LRRK2 |  |  |  |
| KIR2DL4 |  |  |  |
| NUGGC |  |  |  |
| HLA-DRB1 |  |  |  |
| CTSE |  |  |  |
| SIRPB2 |  |  |  |
| GIMAP5 |  |  |  |
| TLR7 |  |  |  |
| HLA-DQA1 |  |  |  |
| SPN |  |  |  |
| GZMM |  |  |  |
| MPEG1 |  |  |  |
| PDCD1LG2 |  |  |  |
| FCGR1BP |  |  |  |
| AKR1B10 |  |  |  |
| CSF2RA |  |  |  |
| HLA-DRB5 |  |  |  |
| SH2D1B |  |  |  |
| RCSD1 |  |  |  |
| CD247 |  |  |  |
| SUCNR1 |  |  |  |
| CD3E |  |  |  |
| FCGR3A |  |  |  |
| HLA-DOA |  |  |  |
| HLA-DRA |  |  |  |
| CARD16 |  |  |  |
| AIF1 |  |  |  |
| NCR3 |  |  |  |
| LST1 |  |  |  |
| PSORS1C2 |  |  |  |
| LILRB3 |  |  |  |
| IGFL2 |  |  |  |
| SLFN12L |  |  |  |
| IGKC |  |  |  |
| IGKV4-1 |  |  |  |
| IGKV3D-20 |  |  |  |
| IGLV4-69 |  |  |  |
| IGLV8-61 |  |  |  |
| IGLV6-57 |  |  |  |
| IGLV1-51 |  |  |  |
| IGLV1-47 |  |  |  |
| IGLV7-46 |  |  |  |
| IGLV5-45 |  |  |  |
| IGLV1-44 |  |  |  |
| IGLV7-43 |  |  |  |
| IGLV1-40 |  |  |  |
| IGLV3-25 |  |  |  |
| IGLV2-23 |  |  |  |
| IGLV3-21 |  |  |  |
| IGLV3-19 |  |  |  |
| IGLV2-18 |  |  |  |
| IGLV2-14 |  |  |  |
| IGLV2-11 |  |  |  |
| IGLV3-10 |  |  |  |
| IGLV3-9 |  |  |  |
| IGLV3-1 |  |  |  |
| IGLC2 |  |  |  |
| IGLC3 |  |  |  |
| IGLC7 |  |  |  |
| TRGC1 |  |  |  |
| TRBV5-1 |  |  |  |
| TRBV19 |  |  |  |
| TRBV20-1 |  |  |  |
| TRBC1 |  |  |  |
| TRBV28 |  |  |  |
| TRBC2 |  |  |  |
| IGHA2 |  |  |  |
| IGHG4 |  |  |  |
| IGHG2 |  |  |  |
| IGHA1 |  |  |  |
| IGHG1 |  |  |  |
| IGHG3 |  |  |  |
| IGHM |  |  |  |
| IGHV1-2 |  |  |  |
| IGHV3-11 |  |  |  |
| IGHV3-15 |  |  |  |
| IGHV1-18 |  |  |  |
| IGHV3-21 |  |  |  |
| IGHV3-23 |  |  |  |
| IGHV1-24 |  |  |  |
| IGHV2-26 |  |  |  |
| IGHV4-28 |  |  |  |
| IGHV3-33 |  |  |  |
| IGHV4-34 |  |  |  |
| IGHV4-39 |  |  |  |
| IGHV1-46 |  |  |  |
| IGHV3-48 |  |  |  |
| IGHV3-49 |  |  |  |
| IGHV5-51 |  |  |  |
| IGHV3-53 |  |  |  |
| IGHV4-61 |  |  |  |
| IGHV1-69 |  |  |  |
| IGHV3-73 |  |  |  |
| UBD |  |  |  |
| MUC5AC |  |  |  |
| APOL6 |  |  |  |
| HLA-DPB1 |  |  |  |
| IGHV4-59 |  |  |  |
| PELATON |  |  |  |
| IGHV3-74 |  |  |  |
| GBP1P1 |  |  |  |
| IGHV3-72 |  |  |  |
| LGALS17A |  |  |  |
| FAM30A |  |  |  |
| LTA |  |  |  |
| TRGC2 |  |  |  |
| ENSG00000227766 |  |  |  |
| HLA-U |  |  |  |
| HLA-DRB6 |  |  |  |
| CXCR2P1 |  |  |  |
| ANKRD36BP2 |  |  |  |
| HLA-DPA1 |  |  |  |
| IGHV4-31 |  |  |  |
| IGHV3-43 |  |  |  |
| HLA-DQB2 |  |  |  |
| PTPRN2-AS1 |  |  |  |
| LINC01871 |  |  |  |
| HLA-DQA2 |  |  |  |
| OR2I1P |  |  |  |
| LINC00426 |  |  |  |
| APOBEC3G |  |  |  |
| IGKV1-6 |  |  |  |
| IGKV3-20 |  |  |  |
| LILRA2 |  |  |  |
| PSMB9 |  |  |  |
| IGKV1-17 |  |  |  |
| TNFRSF13B |  |  |  |
| IGKV1-16 |  |  |  |
| IGKV2-24 |  |  |  |
| IGKV3-11 |  |  |  |
| IGKV1-9 |  |  |  |
| IGHJ3 |  |  |  |
| IGKV1-5 |  |  |  |
| IGKV3-15 |  |  |  |
| LILRA6 |  |  |  |
| IGKV1-27 |  |  |  |
| FCGR2C |  |  |  |
| LINC00861 |  |  |  |
| PCED1B-AS1 |  |  |  |
| LINC02362 |  |  |  |
| LINC02384 |  |  |  |
| LINC01094 |  |  |  |
| IGHGP |  |  |  |
| SIGLEC14 |  |  |  |
| SIGLEC12 |  |  |  |
| IGLL5 |  |  |  |
| GVINP1 |  |  |  |
| ENSG00000255197 |  |  |  |
| TIFAB |  |  |  |
| LINC02446 |  |  |  |
| USP30-AS1 |  |  |  |
| CLEC5A |  |  |  |
| ENSG00000259834 |  |  |  |
| MMP2-AS1 |  |  |  |
| MRC1 |  |  |  |
| MMP12 |  |  |  |
| ENSG00000266088 |  |  |  |
| TAF5LP1 |  |  |  |
| ENSG00000268027 |  |  |  |
| SLC6A14 |  |  |  |
| SPIB |  |  |  |
| IGHV3-30 |  |  |  |
| CCL5 |  |  |  |
| KLHDC7B-DT |  |  |  |
| MIR223HG |  |  |  |
| CCL4 |  |  |  |
| CCL18 |  |  |  |
| CCL4L2 |  |  |  |
| CCL3L1 |  |  |  |
| CCL3 |  |  |  |
| TRAC |  |  |  |
| TRBV7-9 |  |  |  |
| IGLV2-8 |  |  |  |
| IGHV1-69D |  |  |  |
| LINC01943 |  |  |  |
| TRG-AS1 |  |  |  |

**Table S3 Area Under Curve and C-Index**

| AUC  Model | 1-year | 3-year | 5-year | C-index |
| --- | --- | --- | --- | --- |
| Train Set I | 0.677 | 0.689 | 0.723 | 0.679 |
| Test Set I | 0.675 | 0.762 | 0.751 |  |
| Train Set II | 0.682 | 0.685 | 0.764 | 0.691 |
| Test Set II | 0.511 | 0.683 | 0.755 |  |
| Train Set III | 0.601 | 0.672 | 0.712 | 0.638 |
| Test Set III | 0.823 | 0.694 | 0.748 |  |
| Train Set IV | 0.64 | 0.696 | 0.746 | 0.679 |
| Test Set IV | 0.57 | 0.645 | 0.717 |  |
| Train Set V | 0.595 | 0.685 | 0.72 | 0.655 |
| Test Set V | 0.722 | 0.699 | 0.756 |  |
| Train average | 0.639 | 0.6854 | 0.733 | 0.6684 |
| Test average | 0.6602 | 0.6966 | 0.7454 |  |
| Gene model | 0.624 | 0.686 | 0.736 | 0.665 |
| Comprehensive model | 0.763 | 0.724 | 0.757 | 0.743 |

**Table S4 Chemotherapeutic agents and molecular-targeted drugs**

| Name | r | p | Synonyms | Targets | Target pathway | PubCHEM | Datasets | number of cell lines | Screening site |
| --- | --- | --- | --- | --- | --- | --- | --- | --- | --- |
| BI-2536 | 0.251105116 | 5.07E-09 |  | PLK1, PLK2, PLK3 | Cell cycle | 11364421 | GDSC2 | 923 | SANGER |
| Linsitinib | 0.221579016 | 2.77E-07 | OSI-906, ASP-7487 | IGF1R | IGF1R signaling | 11640390 | GDSC2 | 966 | SANGER |
| Sabutoclax | 0.185814821 | 1.76E-05 | BI-97C1 | BCL2, BCL-XL, BFL1, MCL1 | Apoptosis regulation | 46236925 | GDSC2 | 892 | SANGER |
| NVP-ADW742 | 0.173930478 | 5.97E-05 | NVP ADW742, NVPADW742 | IGF1R | IGF1R signaling | 9825149 | GDSC2 | 958 | SANGER |
| Axitinib | 0.169089095 | 9.60E-05 | AG-13736, Inlyta | PDGFR, KIT, VEGFR | RTK signaling | 6450551 | GDSC2 | 961 | SANGER |
| Tozasertib | 0.166530159 | 0.000122674 | MK 0457,MK-0457,MK-045, VX-680 VX 680 VX-68 | AURKA, AURKB, AURKC, others | Mitosis | 5494449 | GDSC2 | 268 | SANGER |
| Ulixertinib | 0.147030419 | 0.000709959 | BVD-523, VRT752271 | ERK1, ERK2 | ERK MAPK signaling | 11719003, 58641927 | GDSC2 | 740 | SANGER |
| Fulvestrant | 0.14453011 | 0.000876255 |  | ESR | Hormone-related |  | GDSC2 | 717 | SANGER |
| ERK_2440 | 0.136345572 | 0.001705436 | SN1051032892, ERK_2440 | ERK1,ERK2 | ERK MAPK signaling |  | GDSC2 | 717 | SANGER |
| KRAS (G12C) Inhibitor-12 | 0.13233847 | 0.002332913 | GTPL8020 | KRAS (G12C) | ERK MAPK signaling | 73555129 | GDSC2 | 894 | SANGER |
| MK-2206 | 0.129526007 | 0.002892295 | MK 2206, MK2206 | AKT1, AKT2 | PI3K/MTOR signaling | 46930998 | GDSC2 | 968 | SANGER |
| PCI-34051 | 0.11723385 | 0.007056161 | AK298746 | HDAC8, HDAC6, HDAC1 | Chromatin histone acetylation | 24753719 | GDSC2 | 894 | SANGER |
| GSK1904529A | 0.105064582 | 0.015827586 | GSK-1904529A, GSK 1904529A | IGF1R, IR | IGF1R signaling | 25124816 | GDSC2 | 965 | SANGER |
| Vorinostat | 0.096090313 | 0.027400548 | Zolinza, SAHA, suberanilohydroxamic acid, suberoylanilide hydroxamic acid, MK-0683 | HDAC inhibitor Class I, IIa, IIb, IV | Chromatin histone acetylation | 5311 | GDSC2 | 959 | SANGER |
| Uprosertib | 0.094895941 | 0.029389544 | GSK2141795, GSK2141795C, GSK-2141795 | AKT1, AKT2, AKT3 | PI3K/MTOR signaling | 51042438 | GDSC2 | 736 | SANGER |
| Epirubicin | 0.090000767 | 0.038886308 | Ellence, Farmorubicin, IMI-28 | Anthracycline | DNA replication | 41867 | GDSC2 | 958 | SANGER |
| AZD1332 | 0.086924955 | 0.046094241 | SN1061387896 | NTRK1, NTRK2, NTRK3 | RTK signaling |  | GDSC2 | 717 | SANGER |
| EPZ5676 | -0.091757183 | 0.03521613 | EPZ-5676, Pinometostat | DOT1L | Chromatin histone methylation | 57345410 | GDSC2 | 962 | SANGER |
| Fulvestrant | -0.095300359 | 0.028702623 | Faslodex, ICI-182780 | ESR | Hormone-related | 104741 | GDSC2 | 963 | SANGER |
| Nelarabine | -0.098549477 | 0.02366733 | Atriance, Arranon |  | DNA replication |  | GDSC2 | 716 | SANGER |
| AZD5991 | -0.103102764 | 0.017905383 | SN1049446612, AZD5991 | MCL1 | Apoptosis regulation |  | GDSC2 | 713 | SANGER |
| YK-4-279 | -0.104010461 | 0.016916116 | YK 4-279 | RNA helicase A | Other | 44632017 | GDSC2 | 958 | SANGER |
| WIKI4 | -0.106504767 | 0.014439894 | WIKI-4, WIKI 4 | TNKS1, TNKS2 | WNT signaling | 2984337 | GDSC2 | 957 | SANGER |
| AT13148 | -0.10712396 | 0.013876935 |  | AKT1 | PI3K/MTOR signaling |  | GDSC2 | 954 | SANGER |
| Bortezomib | -0.109705856 | 0.011732457 | PS-341, LDP-341, Velcade | Proteasome | Protein stability and degradation | 387447 | GDSC2 | 957 | SANGER |
| JAK_8517 | -0.109948984 | 0.01154648 | SN1066590414, JAK_8517 | JAK1, JAK2 | Other, kinases |  | GDSC2 | 717 | SANGER |
| Trametinib | -0.111040444 | 0.010743346 | GSK1120212, Mekinist | MEK1, MEK2 | ERK MAPK signaling | 11707110 | GDSC2 | 966 | SANGER |
| PD0325901 | -0.111663913 | 0.010307151 | PD-0325901, PD 0325901 | MEK1, MEK2 | ERK MAPK signaling | 9826528 | GDSC2 | 968 | SANGER |
| MK-8776 | -0.111999696 | 0.010078802 | SCH900776 | CHEK1, CHEK2, CDK2 | Cell cycle | 16224745 | GDSC2 | 952 | SANGER |
| MN-64 | -0.115461146 | 0.007974058 |  | TNKS1, TNKS2 | WNT signaling | 2802462 | GDSC2 | 894 | SANGER |
| Staurosporine | -0.11622599 | 0.007565692 |  | Broad spectrum kinase inhibitor | RTK signaling | several | GDSC2 | 968 | SANGER |
| Zoledronate | -0.120032429 | 0.005798556 | Zoledronic acid, Zometa, Reclast |  | Other |  | GDSC2 | 717 | SANGER |
| GSK343 | -0.121468481 | 0.005234883 | GTPL8240, AOB3680, AK175558 | EZH2 | Chromatin histone methylation | 71268957 | GDSC2 | 893 | SANGER |
| Cyclophosphamide | -0.121769362 | 0.005123232 | Cytoxan, Neosar, Cyclophosphamid, Procytox, Cyclophosphane | Alkylating agent | DNA replication | 2907 | GDSC2 | 957 | SANGER |
| BMS-754807 | -0.126599048 | 0.003601717 | BMS-754807 | IGF1R, IR | IGF1R signaling |  | GDSC2 | 949 | SANGER |
| LGK974 | -0.13413211 | 0.002029746 | LGK-974 | PORCN | WNT signaling | 46926973 | GDSC2 | 964 | SANGER |
| Ibrutinib | -0.134389059 | 0.001989398 | PCI-32765, CRA-032765, PCI-32765-00 | BTK | Other, kinases | 24821094 | GDSC2 | 890 | SANGER |
| Dasatinib | -0.135302873 | 0.001851779 | BMS-354825-03, BMS-354825, Sprycel | ABL, SRC, Ephrins, PDGFR, KIT | Other, kinases | 3062316 | GDSC2 | 962 | SANGER |
| ZM447439 | -0.135501589 | 0.001823031 | ZM-447439, ZM 447439 | AURKA, AURKB | Mitosis | 9914412 | GDSC2 | 944 | SANGER |
| Venetoclax | -0.136567006 | 0.001675757 | ABT-199, Veneclexta, GDC-0199 | BCL2 | Apoptosis regulation | 49846579 | GDSC2 | 958 | SANGER |
| AMG-319 | -0.137132548 | 0.001602094 | AMG319 | PI3K (beta sparing) | PI3K/MTOR signaling | 68947304 | GDSC2 | 950 | SANGER |
| PFI3 | -0.143570231 | 0.000949156 | PFI-3, PFI 3, AOB2221 | Polybromo 1, SMARCA4, SMARCA2 | Chromatin other | 78243717 | GDSC2 | 894 | SANGER |
| Osimertinib | -0.144647921 | 0.000867673 | AZD9291, AZD 9291, AZD-9291, Tagrisso, Mereletinib | EGFR | EGFR signaling | 71496458 | GDSC2 | 957 | SANGER |
| LJI308 | -0.1461744 | 0.000763284 |  | RSK2, RSK1, RSK3 | PI3K/MTOR signaling | 118704762 | GDSC2 | 954 | SANGER |
| RVX-208 | -0.154576472 | 0.000368694 | 1044870-39-4, Apabetalone | BRD4 | Chromatin other | 24871506 | GDSC2 | 893 | SANGER |
| AGI-5198 | -0.157225083 | 0.000290842 | IDH-C35 | IDH1 (R132H) | Metabolism | 56645356 | GDSC2 | 958 | SANGER |
| BPD-00008900 | -0.157347861 | 0.000287635 |  |  | Other |  | GDSC2 | 743 | SANGER |
| PF-4708671 | -0.161018389 | 0.000205721 | PF 4708671, PF4708671 | S6K1 | PI3K/MTOR signaling | 51371303 | GDSC2 | 268 | SANGER |
| Erlotinib | -0.164174236 | 0.00015332 | Tarceva, RG-1415, CP-358774, OSI-774, Ro-508231, R-1415 | EGFR | EGFR signaling | 176870 | GDSC2 | 955 | SANGER |
| Savolitinib | -0.167581035 | 0.000110951 | AZD6094, Volitinib, AZD-6094, AZD 6094 | MET | RTK signaling | 68289010 | GDSC2 | 957 | SANGER |
| GSK2606414 | -0.180083712 | 3.21E-05 | AK175551 | PERK | Metabolism | 53469448 | GDSC2 | 886 | SANGER |
| Sapitinib | -0.184335208 | 2.06E-05 | AZD8931 | EGFR, ERBB2, ERBB3 | EGFR signaling | 11488320 | GDSC2 | 966 | SANGER |
| GSK591 | -0.184709292 | 1.98E-05 | EPZ015866, GSK3203591 | PMRT5 | Chromatin histone methylation | 117072552 | GDSC2 | 954 | SANGER |
| IWP-2 | -0.185391708 | 1.84E-05 | Wnt Inhibitor IWP-2 | PORCN | WNT signaling | 2155128 | GDSC2 | 893 | SANGER |
| AZD8055 | -0.188167081 | 1.37E-05 | AZD-8055 | MTORC1, MTORC2 | PI3K/MTOR signaling | 25262965 | GDSC2 | 745 | SANGER |
| AZ960 | -0.189969511 | 1.13E-05 | AZ 960, AZ-960 | JAK2, JAK3 | Other, kinases | 25099184 | GDSC2 | 892 | SANGER |
| Temozolomide | -0.190817515 | 1.03E-05 | Temodar, Temodal, M-39831, SCH 52365 | DNA alkylating agent | DNA replication | 5394 | GDSC2 | 965 | SANGER |
| CZC24832 | -0.205835398 | 1.89E-06 | GTPL6653 | PI3Kgamma | PI3K/MTOR signaling | 42623951 | GDSC2 | 894 | SANGER |
| AZD3759 | -0.217219771 | 4.78E-07 |  | EGFR | EGFR signaling | 78209992 | GDSC2 | 966 | SANGER |
| Entospletinib | -0.223936075 | 2.05E-07 | GS-9973 | SYK | Other, kinases | 59473233 | GDSC2 | 894 | SANGER |
| IAP_5620 | -0.2330454 | 6.24E-08 | SN1043546339 | IAP | Other |  | GDSC2 | 714 | SANGER |
| Selumetinib | -0.271511683 | 2.33E-10 | SN1103949345, AZD1480 | MEK1, MEK2 | ERK MAPK signaling |  | GDSC2 | 717 | SANGER |
| NU7441 | -0.277896189 | 8.43E-11 | KU-57788, NU-7432, NU-7741 | DNAPK | Genome integrity | 11327430 | GDSC2 | 945 | SANGER |
| Ruxolitinib | -0.312605463 | 2.07E-13 | INCB-18424, Ruxolitinib Phosphate, Jakafi | JAK1, JAK2 | Other, kinases | 25126798 | GDSC2 | 898 | SANGER |
| XAV939 | -0.316293882 | 1.04E-13 | NVP-XAV939, XAV-939, XAV 939 | TNKS1, TNKS2 | WNT signaling | 2726824 | GDSC2 | 893 | SANGER |
| SB216763 | -0.340376755 | 9.24E-16 | SB-216763, SB 216763 | GSK3A, GSK3B | WNT signaling | 176158 | GDSC2 | 956 | SANGER |
| Ribociclib | -0.358839871 | 1.85E-17 | LEE011, NVP-LEE011, LEE011-BBA | CDK4, CDK6 | Cell cycle | 44631912 | GDSC2 | 959 | SANGER |
| AZD6482 | -0.401603142 | 7.63E-22 | AZD 6482, AZD-6482, AK-55409 | PI3Kbeta | PI3K/MTOR signaling | 44137675 | GDSC2 | 267 | SANGER |
| KU-55933 | -0.526154968 | 7.37E-39 | KU55933 | ATM | Genome integrity | 5278396 | GDSC2 | 955 | SANGER |
